# Spatial filters of function and phylogeny determine morphological disparity with latitude

**DOI:** 10.1371/journal.pone.0221490

**Published:** 2019-08-29

**Authors:** K. S. Collins, S. M. Edie, T. Gao, R. Bieler, D. Jablonski

**Affiliations:** 1 Department of the Geophysical Sciences, University of Chicago, Chicago, Illinois, United States of America; 2 Committee on Computational and Applied Mathematics, Department of Statistics, University of Chicago, Chicago, Illinois, United States of America; 3 Integrative Research Center, Field Museum of Natural History, Chicago, Illinois, United States of America; Nanjing Agricultural University, CHINA

## Abstract

The drivers of latitudinal differences in the phylogenetic and ecological composition of communities are increasingly studied and understood, but still little is known about the factors underlying morphological differences. High-resolution, three-dimensional morphological data collected using computerized micro-tomography (micro-CT) allows comprehensive comparisons of morphological diversity across latitude. Using marine bivalves as a model system, this study combines 3D shape analysis (based on a new semi-automated procedure for placing landmarks and semilandmarks on shell surfaces) with non-shape traits: centroid size, proportion of shell to soft-tissue volume, and magnitude of shell ornamentation. Analyses conducted on the morphology of 95% of all marine bivalve species from two faunas along the Atlantic coast of North America, the tropical Florida Keys and the boreal Gulf of Maine, show that morphological shifts between these two faunas, and in phylogenetic and ecological subgroups shared between them, occur as changes in total variance with a bounded minimum rather than directional shifts. The dispersion of species in shell-shape morphospace is greater in the Gulf of Maine, which also shows a lower variance in ornamentation and size than the Florida Keys, but the faunas do not differ significantly in the ratio of shell to internal volume. Thus, regional differences conform to hypothesized effects of resource seasonality and predation intensity, but not to carbonate saturation or calcification costs. The overall morphological differences between the regional faunas is largely driven by the loss of ecological functional groups and family-level clades at high latitudes, rather than directional shifts in morphology within the shared groups with latitude. Latitudinal differences in morphology thus represent a complex integration of phylogenetic and ecological factors that are best captured in multivariate analyses across several hierarchical levels.

## Introduction

The most fundamental observation on biodiversity is that it is not uniform across the globe [1–3]. The dominant pattern for most clades is the latitudinal diversity gradient (LDG), the stepwise reduction in taxon numbers [4–5] and ecological functional groups [6–7] from tropics to poles. LDG patterns have been identified in many ordinal or categorical aspects of biology, but morphological diversity is inherently continuous and multivariate, requiring methods beyond identification and enumeration to quantify at large spatial scales. We use an extensive multivariate morphological dataset in a biological group that has become a model macroecological and macroevolutionary system—the marine bivalves—to extend analysis of the morphological LDG beyond size parameters alone. Marine bivalves show strong gradients in taxonomic and functional diversity from equator to poles [7–9], their shell is homologous across all species and provides much information on their mode of life. Further, they are major ecosystem engineers, acting as reef-builders and water-quality enhancers, and constitute a major source of protein for billions of humans worldwide [10], so that their responses to the abiotic environment and its accelerating changes [2,11] are of more than academic interest.

The bivalve shell is an ideal vehicle for studying spatial patterns in phylogeny and morphology. Taxonomy derived from shell traits generally reflects the current understanding of phylogeny, with families representing stable, monophyletic clades [12]. The shell also provides a summary of whole-body morphology, where the internal surface of the shell protects the soft parts of the animal, and the external surface interacts with the surrounding environment in such a way that its overall configuration broadly reflects the animal’s habits [13–14]. The shape of the shell interior records the overall configuration of the soft tissues, anteroposterior and dorsoventral elongation, and other functionally significant attributes, so that increases or decreases in the variety of shell shapes reflect the gain or loss of modes of life at greater resolution than categorical classifications of function. Functional groups are, therefore, largely composed of species with similar phenotypes. Some widely distributed functional groups have especially high species richness, a condition referred to as stacking when they co-occur geographically, which is thought to reflect a shared mode of life that either exploits particularly abundant resources or has enjoyed long-term habitat stability promoting finer niche partitioning [7,15]. Such fine niche partitioning may be reflected in differences in shell size, shape, ornamentation and thickness, all of which influence burrowing ability and rate [13] and defense against predation [16–17]. Larger, thicker or more elaborate shells require more resources and energy, which may be limiting at higher latitudes, where food resources are more seasonal [15, 18] and the ocean has lower carbonate saturation states [19].

**Fig 1:**
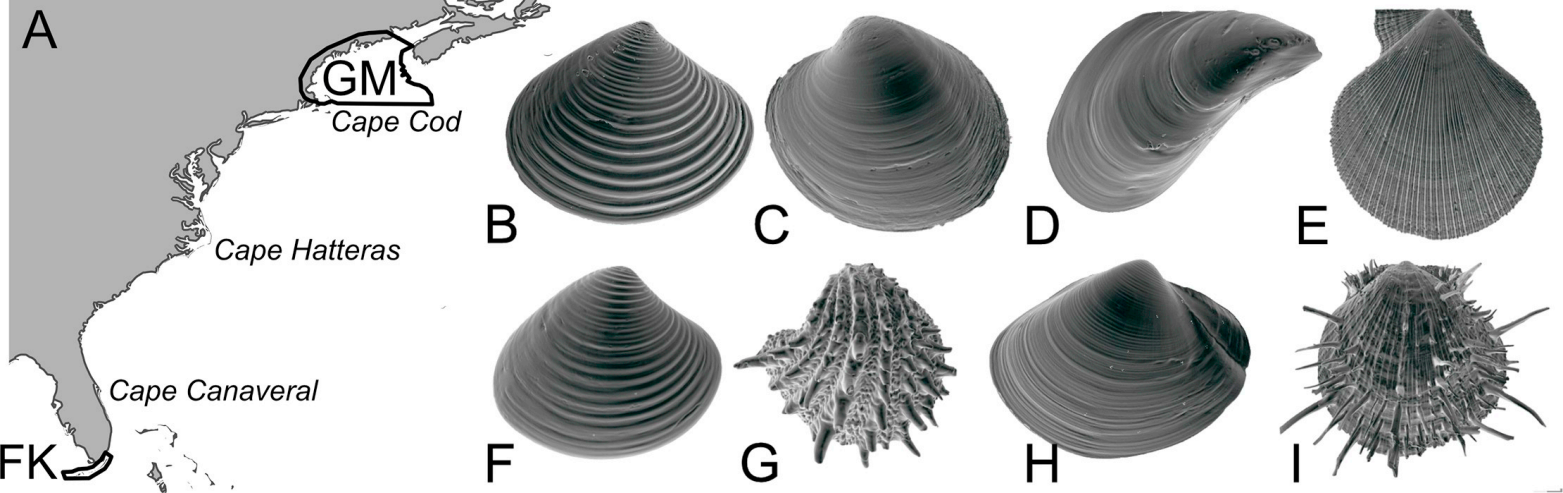
Geographical context and example specimens (not to scale). A: East coast of North America, showing sampled regions (GM = Gulf of Maine, FK = Florida Keys) and three major biogeographic breaks. GM specimens: B: *Astarte borealis* Schumacher, 1817 (Astartidae). C: *Arctica islandica* (Linnaeus, 1767) (Arcticidae). D: *Mytilus edulis* Linnaeus, 1758 (Mytilidae). E. *Chlamys islandica* (Mueller, 1776) (Pectinidae). FK specimens: F: *Astarte subaequilatera* G. B. Sowerby II, 1854 (Astartidae). G: *Arcinella cornuta* Conrad, 1866 (Chamidae). H: *Anatina anatina* (Spengler, 1802) (Mactridae). I: *Spondylus americanus* Hermann, 1781 (Spondylidae).

We quantify changes in taxonomic, functional, and morphological composition from two of the best-studied regional faunas: the Florida Keys (FK; 24.8°N 80.9°W) at the northern edge of the tropical Caribbean Province, and the Gulf of Maine (GM; 42.9°N 70.3°W) at the southern edge of the cold Nova Scotian Province [20], which are separated by three major biogeographic breaks: Cape Canaveral (~28°N), Cape Hatteras (~35°N), and Cape Cod (~42°N) [19, 20] (Fig 1). Taxonomic and functional diversity decline between FK and GM from 355 to 90 species and from 37 to 26 functional groups, respectively. We use microCT scans of 95% of the total number of species known from both regions (N = 424 of 445) to capture the full spectrum of bivalve form in each region and discuss how apparent changes in form are associated with changes in the abiotic and biotic environments of these two regions.

## Methods

### Specimens

One representative adult or near-adult specimen was sampled per species (S1 Table), totaling 95% of the species known from the two faunas, based on literature- and field/museum-based inventories [21–25]. All specimens were sourced from existing museum collections and no additional fieldwork was carried out. All specimens are deposited in public museum collections. Metadata, including museum collection numbers, can be found in S1 Table. Many FK specimens were previously figured [25] and some (or representatives of the same source populations) were used as morphological and molecular exemplars in Bivalve Tree-of-Life (BivAToL) analyses [12,26–27]. For species that occur in both regions (N = 12), one specimen from each region was included. An effort was made to select specimens collected from within the target FK and GM regions, but when suitable shells representing regional species were not readily accessible for scanning purposes, they were represented by conspecific specimens collected elsewhere within the same climate zone (with such localities flagged with † in S1 Table). Species which could not be included at all due to lack of material in collections are listed at the end of S1 Table. Molecular data are not available for most of the species used in this study, so we used family identity as a proxy for phylogenetic relatedness; bivalve families are taxonomically stable and almost all are found to be monophyletic groups in recent molecular analyses [12].

This “one specimen per species” sampling protocol was necessary to achieve as complete a coverage of the faunas as possible within a reasonable timeframe while taking into account museum holdings of species, some of which are very rare. However, where possible, we used additional specimens from other regions outside the study area to quantify intraspecific variation and thus understand the potential biases in representing a species’ morphology with a single individual (N_species_ = 65, N_specimens_ = 156). Levels of intraspecific variation in this dataset fluctuate, but most of the species exhibit only a small range of values among their individuals compared to the total range among all species (S1 Fig).

### Site climate and environment

The site boundaries for summarizing geographic and climatic data for FK and GM are shown in Fig 1. The mean and range of sea surface temperature were summarized within each site using MARPSEC [28], net primary productivity from MODIS [29], and shelf area at < 200 m water depth and coastline length were estimated at the 1:10 m scale using the bathymetry and coastline geometries from Natural Earth (Table 1). Winter and summer aragonite saturation states of the ocean are taken from [30–32].

**Table 1:** Climatic and geographic characteristics of the Florida Keys and Gulf of Maine regions. Values are rounded to be commensurate with the uncertainty in the boundary definitions for these two regions.

	Annual mean sea surface temp. [°C]	Annual range of sea surface temp. [°C]	Annual mean net primary productivity [mg C / m^2^ / day]	Annual range of net primary productivity [mg C / m^2^ / day]	Coastline length [km]	Shelf area [km2]	Summer aragonite saturation state [Ω_arag_]	Winter aragonite saturation state [Ω_arag_]
FK	26	7	1600	3750	450	8,000	4.1	3.7
GM	9	14	1500	2300	2,000	53,000	2.5	1.2

### Scanner

Shells were scanned using a GE v|tome|x scanner with a 240kV micro CT tube, housed in the University of Chicago Paleo-CT facility. Scan resolutions vary between 8–121 microns per voxel (mean = 43 microns per voxel). Full scan parameters are associated with individual Morphosource files (see below). One valve was scanned for equivalve taxa (in which the valves are essentially mirror images of each other), and both valves were scanned for inequivalve taxa. Surfaces were fit to the voxel data from the CT scans and output as ‘.stl’ mesh files. Isolated parts were removed from each mesh, debris was digitally removed (e.g. sand, epibionts, worm/sponge borings, etc.), and surfaces were made manifold prior to analyses. The cleaned surface meshes are available for download on request from https://www.morphosource.org/Detail/ProjectDetail/Show/project_id/692. Researchers interested in our mesh processing methods are encouraged to contact the first authors for more details. The data and R code for generating all figures and data tables are provided as supplementary data files S1 and S2 respectively.

### Morphological features

#### Proportion of the animal that is shell (shell proportion)

**Fig 2:**
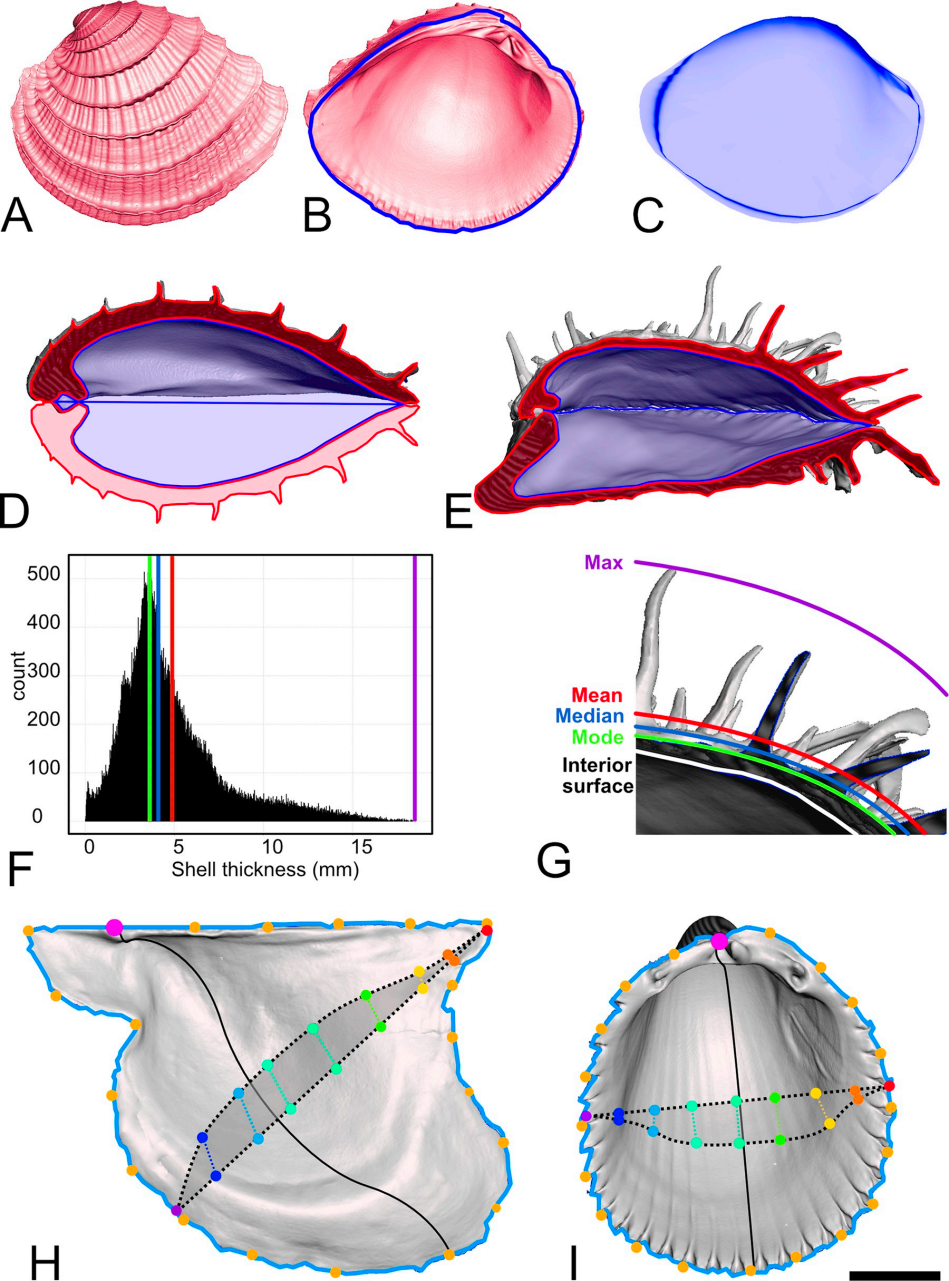
Shell proportion and maximum ornamentation parameters. A: Left valve of *Chione elevata* (Say, 1822), external view. B: Internal view, with blue line indicating the landmarked commissure. C: The internal volume (IV) of the interior surface bounded by the commissure. D: Cross-section of the valve from panels A and B illustrating the calculation of total shell volume (TSV; red) and total internal volume (TIV; blue) from an equivalve shell by duplication of values. E: Cross-section of both valves of *Spondylus americanus* Hermann, 1781, illustrating calculation of TSV (red) and TIV (blue) from the addition of inequivalve shell volumes. (NB: for clarity, only 2D sections are colored to indicate shell vs. internal volume, but actual calculations are performed in 3D). F: Histogram of 100,000 thickness measurements calculated from the top valve of the shell shown in panel E. The white line is the internal surface, the green line the modal thickness, the blue line the median, and the red line the mean. The maximum thickness recorded from this shell is indicated by the purple line. G: Close-up cross-section of part of the top valve from panel E, with lines added to illustrate the relationship between the summary statistics from panel F and the shell scan itself. H: Illustration of semilandmark placement on *Pteria colymbus* (Röding, 1798) (Pteriomorpha; Pteriidae), and I: *Trachycardium egmontianum* (Shuttleworth, 1856) (Imparidentia; Cardiidae). (Scale bar = 1cm, for both shells). For clarity, only illustrative elements of the landmarking scheme are shown. The blue curve and yellow points indicate the manually landmarked commissure curve used to split the shell into interior and exterior mesh surfaces, with the pink point as the point on the commissure closest to the beak. The solid black line indicates the intersection of the plane placed through the point closest to the beak and the 50% point of the commissure curve. The dashed line indicates the intersection of a plane perpendicular to the solid line with the mesh. Points that intersect the mesh and the plane are projected to a flat line in the plane of the shell commissure, sorted based on cumulative Euclidean distance to the anteriormost point, and then re-projected to the mesh surface; e.g. in this example, 10 semilandmarks are placed at 10% distance intervals at the 50% distance along the dorso-ventral sampling axis. The rainbow-colored points along the dashed lines in panels H and I are considered operationally homologous for the purposes of generalized Procrustes analysis in the same way that semilandmarks slid around a curve to minimize bending energy are considered homologous in more traditional semilandmarking procedures.

“Shell proportion” is the proportion of the total animal volume that consists of shell material (CaCO_3_ and associated organics) (Fig 2D and 2E). The volumes are calculated as the sum of signed tetrahedra volumes across a surface mesh [33], which gives the volume of shell material produced by the animal. The soft-tissue+water volume (‘internal volume’) of the animal is found by centering the mesh surface of the shell interior on the origin and taking the sum of signed tetrahedra volumes. For equivalve taxa, the shell volume and internal volumes are each multiplied by two to calculate the total shell volume (TSV) and total internal volume (TIV). For inequivalve taxa, the shell volume and internal volume of each valve of the individual are summed, respectively, to calculate the TSV and TIV. Shell proportion (propSV) is then calculated as TSV / (TSV + TIV). In the few taxa which have either a posterior or pedal gape, this approach underestimates TIV, as it does not include their non-retractable tissues, but it always includes the volume required to house vital organs.

#### Body size

We use centroid size (the square root of the sum of squared distances of each point on a mesh from the centroid of that mesh) as our size metric. Since this metric is sensitive to the number of points, meshes are first standardized to 100,000 equally spaced points on the mesh surface using a strict Poisson-disk subsampling in the R package “Rvcg” [34]. For equivalve taxa, we multiply the centroid size of a single valve by two and for inequivalve taxa, we sum the centroid sizes of the two valves. Centroid size is uncorrelated with shape, but is highly correlated to 2D size measures [35], and thus our results are comparable to previous bivalve size studies.

#### Ornamentation

We treat a shell’s ornamentation as a deviation (by addition of material) from a hypothetically smooth surface of the shell (such as the remaining surface after the abrasion experiments of Stanley [36]). We designed a semi-automated approach to define the hypothetically smooth surface of the shell and then measure a shell’s ornamentation as follows:

1. Spilt each mesh into interior and exterior surfaces along the commissure (blue line in Fig 2B).
2. Place 100,000 equally spaced points on each surface using a strict Poisson-disk subsampler.
3. Calculate the minimum Euclidean distance between each point in the exterior point cloud to the interior point cloud, which yields 100,000 measurements of minimum shell thickness over the entire shell.
4. Bin these thickness measurements into 100 equally spaced intervals. The mean value of the interval that contains the most values (the modal interval) is used as the baseline thickness of the shell. Projecting this distance from each point lying on the shell interior then serves as the surface of the hypothetically smooth shell.
5. Ornamentation values are then calculated as the difference between the distance of the hypothetically smooth shell surface and the distance from the interior surface to the exterior surface. The maximum value distance is retained as the ‘maximum ornamentation height’. Subsequent analyses were not sensitive to using the absolute maximum or the 99^th^, 98^th^, or 95^th^ quantiled value.

The strength of the ornamentation on a shell determines the skew of the distribution of thickness measurements used for estimating the hypothetically smooth shell surface. *Arcuatula papyria* (Conrad, 1846) is almost completely smooth and has an approximately Gaussian distribution of shell thickness measurements. In contrast, a specimen of *Spondylus americanus* (Fig 2E and 2G) has a highly skewed distribution of shell thickness measurements because its spines are large (sometimes >25 mm) but infrequent features compared the majority of the shell surface. Thus, the modal value of shell thickness measurements, not the median or mean, tends to best represent the distance to the hypothetically smooth shell surface. This approach provides our current best approximation to Stanley’s un-ornamented shell surface [36] in a time-efficient and reproducible manner.

#### Internal shell shape

We analyzed the shape of the shells in the dataset using semilandmark geometric morphometrics. Bivalves are limited in the number of discrete biologically homologous points, or Type 1 landmarks (*sensu* Bookstein [37]), that can be located across the entire class—the only truly homologous anatomical point present in all shells is the apex of the beak in the embryonic or larval shell (prodissoconch), and this feature may be hidden if the adult shell (dissoconch) coils too tightly or lost if the specimen is very worn. However, the shell overall is a homologous structure among animals, so we placed semilandmarks across the internal surface of each shell to describe its form in the following manner adapted from the “eigensurface” approach of Polly and MacLeod [38]. This is a conservative approach to quantifying shape differences because semilandmark methods applied to simple shapes with high similarity between the most disparate individuals tends to weaken the ability to detect shape differences [39].

1. The interior surface of a shell (a triangular mesh, e.g. Fig 2H and 2I) is scaled to the centroid size of a 75-point commissure curve (points subsampled from the same manually defined commissure curve discussed under ‘ornamentation’ and represented as the blue outlines in Fig 2B, 2H and 2I). We normalized shape data by centroid size rather than shell volume because it provides a more intuitive size gradient for bivalves. For example, in this dataset, normalizing by volume would lead to thinner shelled animals (e.g. *Ensis directus*) scaling to relatively larger sizes than thicker shelled animals of approximately the same area (S10 Fig)—a common way of understanding bivalve body size [35].
2. The beak point, anterior point, ventral point, and posterior point along the commissure curve are defined as the points 0%, 25%, 50%, and 75% of the clockwise cumulative Euclidean distance along the curve starting at the point closest to the beak of the shell (pink point Fig 2H).
3. These points are then used to rotate the mesh to a square defined by the XY plane by minimizing the sum of squared Euclidean distances from the beak point to (0,0,0), the anterior point to (1,1,0), the ventral point to (1,0,0), and the posterior point to (0,1,0).
4. If the valve is a “right” valve, then the mesh is mirrored across the XY plane to project the mesh as a “left” valve, which is the operational valve for all downstream shape analyses.
5. After translation and rotation to a common reference frame (the XY plane described above), a sampling axis is defined by the vector originating at the beak point and terminating at the ventral point, with sampling points set incrementally at 1% of the magnitude of that vector (i.e. at 1%, 2%, 3% … 98%, 99%, 100% of the vector magnitude).
6. For each sampling point along the sampling vector, a sampling plane is defined as the unique plane passing through the sampling point that is perpendicular to the sampling vector. Each sampling plane is intersected with the edges of the triangular mesh to obtain a collection of intersected points for each sampling point on the sampling vector.
7. Intersected points were sorted relative to the anteriormost point by first projecting them onto the XY plane (i.e. setting the Z coordinate to 0), then computing the Euclidean distances between each projected point and the “reference” anteriormost point, and finally sorting the projected points based on increasing projected Euclidean distances to the reference point. After the sort, the Euclidean distances between each projected intersecting point and the reference point are monotonically increasing. The intersecting curve is then subsampled by choosing evenly spaced points, from 0% to 100% by increments of 1% of the Euclidean distance between the reference point and the farthest projected intersecting point. These subsampled projected intersecting points are then re-projected back to the mesh surface by adding back their original Z coordinate components; the resulting subset of the intersecting points are the semi-landmarks determined by the current sampling plane. This “flat-sorting” approach most consistently orders semi-landmarks in a homologous fashion across shells with complex surface features (e.g. spikes and recurved concentric ornamentation), which can produce unstable point sorting based on Euclidean distances in three-dimensional space.
8. This sampling procedure is repeated for all steps along the sampling axis to produce a grid of semi-landmarks across the mesh surface (N points = 10,100). All specimens were scaled and aligned using Procrustes superimposition on the “fixed” landmarks of each shell (i.e. the beak point, anterior point, ventral point, and posterior point). The gridded semi-landmarks of each shell were then rotated into the Procrustes configuration.

### Functional groups

We assigned bivalve genera to single states across four functional axes [7, 40]: feeding method, fixation to substratum, mobility, and substratum use, and combined the four to produce functional group classifications. Abbreviations for functional groups used in text and figures are in Table 2.

**Table 2:** Key to functional group abbreviations.

Mobility	Fixation	Substratum	Feeding
IM–Immobile	UN–Unattached	BO–Borer	CA–Carnivorous
MO–Mobile	BY–Byssate	DIS–Deep-infaunal siphonate	CH—Chemosymbiotic
SW–Swimming	CE–Cemented	EP–Epifaunal	MDS–Mixed deposit/suspension
		INA–Infaunal asiphonate	SBD–Subsurface deposit
		NE–Nestler	SRD–Surface deposit
		SMI–Semi-infaunal	SUS–Suspension
		SIS–Shallow-infaunal siphonate	

### Analyses

**Fig 3:**
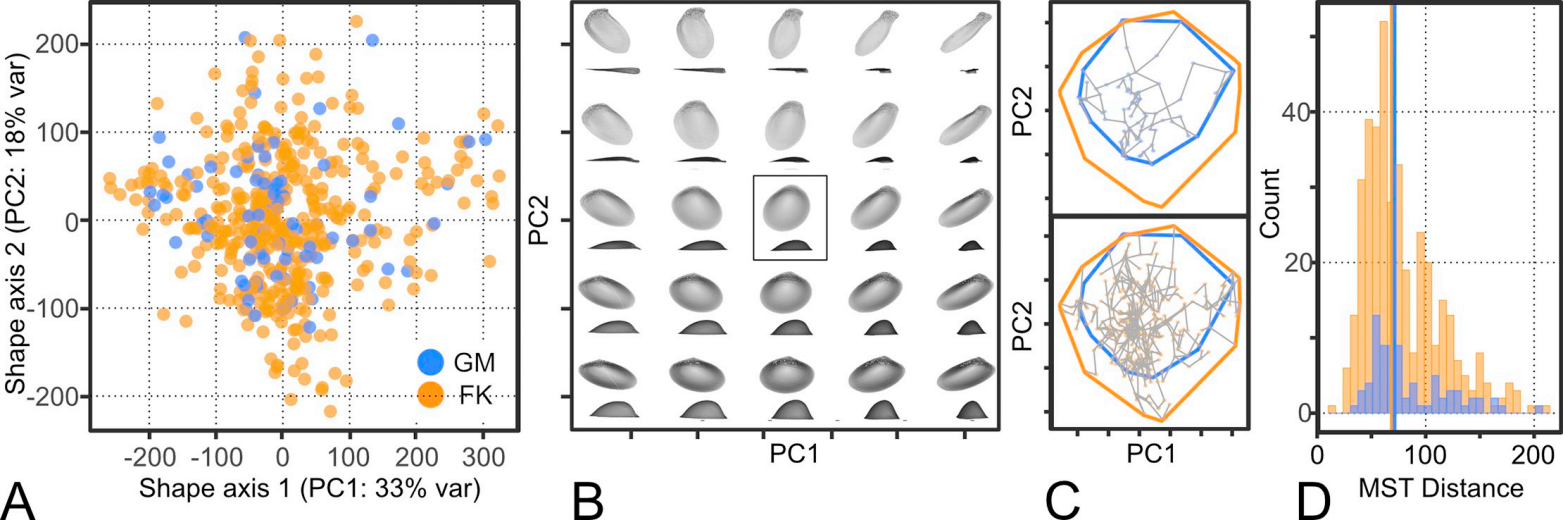
Internal shape space. A: Morphospace of first two principal component axes, for illustrative purposes. B: Projected shell shapes within the PC 1–2 subspace. Each simulated 3D shape is shown by a pair of images, approximating standard illustrations of shells: the uppermost oriented to ‘height/length’ and the lowermost oriented to ‘length/width’. The mean shape is enclosed in a box. C (upper): The morphospace showing only GM points, joined by their minimum spanning tree estimated across PCs 1–23. The convex hulls for both faunas are plotted. C (lower): As for upper, but points and MST are for FK. D: Histogram of distances from the MSTs, colored by region.

Principal Components Analysis (PCA) was used to create a morphospace of the Procrustes-aligned coordinates describing the shape of the interior shell surface. A power analysis of internal shell shape reveals that 23 PCs adequately capture the variation in shell shape for this dataset (S9 Fig). For illustrative purposes, the variation in shell shape within the subspace of the PCA was visualized by defining a point-grid in the space of PC1-PC2 and using the rotation matrix to back-project the scores along this grid to the original space of landmark coordinates (Fig 3B).

We quantify shell shape changes between FK and GM in the morphospace defined by PC axes 1–23 (total 95% cumulative percent of the explained variance) using morphological disparity (sums of variances [40–43]), the median minimum spanning tree distance for each subgroup [44] and an approximation of total morphospace occupation: the cumulative sum of ranges in PC scores across axis 1–23 (S4 and S6 Figs). Convex hulls are commonly used to describe total morphospace occupation, but application to greater than 10 dimensions become computationally intractable, hence our use of cumulative sums of ranges instead. Only families and functional groups with >2 species in both regions were included in these comparisons.

We used pairwise Wilcoxon tests (with a Bonferroni correction) to test for differences in disparity between the Gulf of Maine and Florida Keys faunas, and then between the faunas subset by family or functional group (S2 Table). The Bonferroni correction attempts to minimize false positives and may therefore increase false negatives, meaning that our results are likely to be conservative with respect to identifying statistically significant differences in disparity. To examine statistical differences in dispersion (median minimum spanning tree distance) and morphospace occupation (cumulative sum of ranges in PC scores across axes), we reshuffled the regional identities of species and re-ran each measure 1000 times to derive an expected mean and 95% confidence interval (S4 and S6 Figs). To test for differences in the distributions of observed MST distances between regions for the whole fauna and for each family or functional group, we also used Kolmogorov-Smirnov tests (S2 Table).

Changes or constancy between regions in the values of ornamentation, size, and shell proportion were assessed using the method of Jablonski [45]. Each variable in the trait-space is scaled, but not centered, so that changes in these morphological values are of comparable magnitude (i.e. unit changes in standard deviations). The minimum observed value for each subgroup (whole-fauna, family, or functional group) in the Gulf of Maine is then subtracted from the minimum observed value for the subgroup in the Florida Keys to produce the change in minimum value, and the same is done for the maximum observed values to find the change in maximum value between regions. Subgroups are then plotted in a bivariate space where the X axis is the change in minimum and the Y axis is the change in maximum. The bivariate space is divided into nine fields using the first positive and negative standard deviations of difference values from the mean for each axis. Values within one standard deviation of the mean change are interpreted as not showing any interpretable difference in morphology between regions.

## Results

### Shape

The PCA of the Procrustes-aligned semi-landmark configurations explained 33% and 18% of the total variation along the first two axes, respectively (Fig 3A, 3B and 3C), with 95% of the total variation explained by PCs 1–23 (S9 Fig). The GM fauna’s morphospace is smaller than that of the FK fauna, and smaller than expected by random reshuffling of regional identities, largely due to the loss of families scoring low on PC 2, e.g. Arcidae (ark shells) and Corbulidae (basket clams) (Fig 3A, 3B and 3C, S2 Table, S4 and S8 Figs). Within the high-dimensional morphospace (PCs 1–23), the taxa are more widely dispersed such that the median minimum spanning tree distance is greater in GM than in FK, but disparity (the sum of squared distances between each species and the regional mean) is equal. These differences in dispersion and disparity between regions are also present in the lower dimensional morphospace (PCs 1–2).

Nine families and eight functional groups have sufficient species richness in both regions (N>3 species) to examine morphospace dynamics with latitude. Of the nine families, three show smaller morphospace occupation in GM compared to FK (the Cardiidae [cockles], Mactridae [surf clams], and Pectinidae [true scallops]), four exhibit lower stacking in GM compared to FK (the Mytilidae [true mussels], Thraciidae, Tellinidae, and Veneridae [venus shells]), and two are unchanged between regions (the Astartidae and Nuculidae [nut clams]) (S2 Table, S2 and S5 Figs). Of the eight functional groups, two show smaller morphospace occupation in GM relative to FK, five show lower stacking in GM compared to FK, and one shows little to no change (S2 Table, S3 and S6 Figs). The twelve species shared by the two regions show no consistent changes in shell shape with latitude along any of the 23 PC axes comprising the interior shell shape morphospace (S7 Fig).

### Size and ornamentation

**Fig 4:**
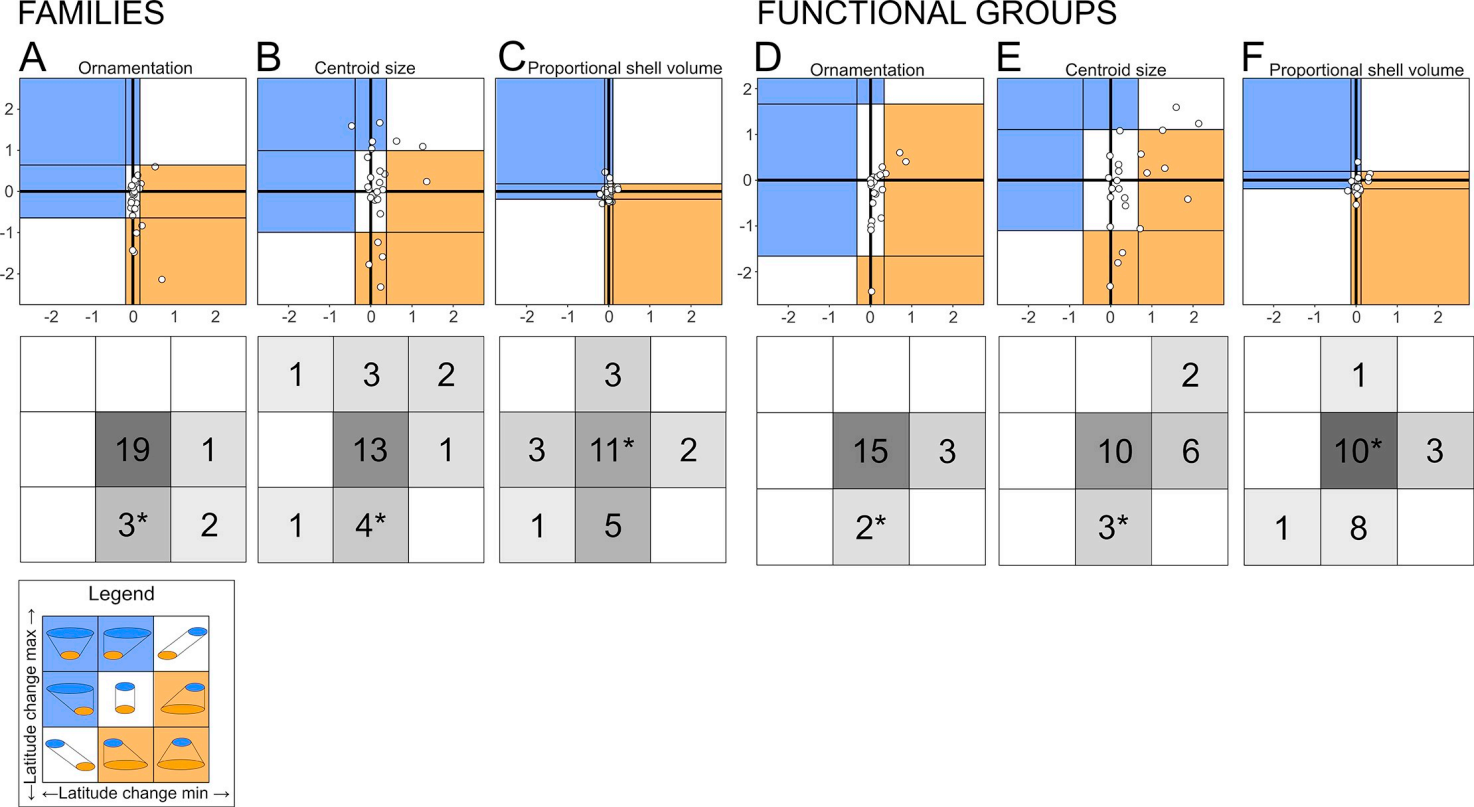
Changes in trait ranges. The upper plot in each panel is divided using standard deviations from the mean change into nine fields characterizing a different pattern of range change with latitude (see legend). Panels A, B, C show families, Panels D, E, F show functional groups. In the upper plots, blue fields = GM fauna has a greater range, orange fields = FK fauna has a greater range, and white fields = no change in faunal range. Points are only plotted for groups having >2 species in both regions. Shading density in lower plots is proportional to number of subgroups in sector; * = position of entire fauna.

Shell size and ornamentation do not show a simple directional change from FK to GM. Instead, both morphological values show a contraction in their range of variation via decreased maxima and bounded minima (Fig 4A, 4B, 4D and 4E, lower plots, fields with asterisks). Decreases in the maximum values of ornamentation are underlain both by the loss of entire families and by a decrease in family-level maxima in 5 of the shared families, with the remaining 20 shared families showing no change in maxima (Fig 4A and 4D, upper plots). Shell size also shows no change in minimum values between the regions and a decrease in maximum values owing to the loss of entire families, but maximum values within shared families show no systematic change (Fig 4B and 4E, upper plots). The larger maximum body sizes and higher ornamentation values in FK derive from tropics-only cemented epifaunal suspension feeders—Chamidae (jewel-boxes), Gryphaeidae (honeycomb oysters), Plicatulidae (kittenpaws), and Spondylidae (thorny oysters) (S1 File). Only Ostreidae (true oysters) represent this functional group in GM, where they are less-ornamented than in FK. Again, the twelve species shared by the two regions show no consistent pattern of change in size or ornamentation (S7 Fig).

### Shell proportion

For species within these two faunas, the shell comprises 6–80% of the total animal volume with most species falling between 20–40% (the inter-quartile range; S1 File). Unlike ornamentation and size, shell proportion shows no overall change in minimum or maximum values from FK to GM (Fig 4C and 4F, lower plots, fields with asterisks). The same is true of most families and functional groups shared between the regions (Fig 4C and 4F) and for the twelve species shared by the two regions (S7 Fig). This morphological value shows no systematic change in either minimum or maximum values, although the most common within-family pattern is constancy of lower and upper bounds, with the next most common being a contraction in the range of variation with a bounded minimum (Fig 4C), as seen in body size and ornamentation. A few families and functional groups exhibit a smaller range of shell proportion values in GM but with a fixed maximum (Fig 4C and 4F), implying that their shells tend to be thicker on average at higher latitudes.

## Discussion

The potential drivers of the LDG are complex and difficult to measure directly, and no single component of biodiversity can fully capture the composite effects of all of the biotic and abiotic factors arrayed along the LDG. Morphological analysis provides fine-scale evidence of the effects of these hard-to-quantify-drivers (i.e. predation pressure and resource abundance) and how they affect the spatial deployment of biodiversity.

### Changes in the environment, species richness, and shell shape

Species richness drops dramatically from FK to GM and functional richness also declines significantly but less severely, reflecting the redundancy of tropical species in functional categories (as seen in other taxa and regions [7, 46–47]). The range of shell shapes also appears to decline from FK to GM. These declines in taxonomic and functional richness, and in shell shape diversity, are inconsistent with a simple expectation from richness-area relationships: GM covers nearly 7 times the habitable shelf area as FK and has ~4 times the length of coastline (Table 1). The lower diversity of shell shapes in GM could result from a random attenuation of the shapes observed in the FK fauna, but the total morphospace occupation of the lower-richness GM fauna is less than expected from a random sampling of the higher-richness FK fauna.

The smaller range of shell shapes in GM compared to FK is also not likely attributable to differences in available habitats or resources. Both regions have varying proportions of softground (sands and muds) and hardground (rocks and biogenic carbonate) habitats, with food resources that support both suspension-feeding and deposit-feeding [25,48]. FK does have coral reefs, which are absent from GM, but this habitat is not associated with particular families, functional groups, or shell shapes on this coastline [25,49]. Thus, the absence of FK-specific forms in GM is probably not tied to the loss of region-specific habitats and resources. Instead, the smaller range of shell forms in GM is related to the loss of inflated shell shapes associated with two specific families, the Arcidae (ark shells) and the Corbulidae (basket clams), neither of which represent a functional group or life habit unique to FK.

Not only is there a smaller total range of shell shapes in FK compared to GM, but the species in the shell shape morphospace are also overdispersed. We interpret this pattern as indicating reduced stacking of similar morphologies in GM, which may be related to decreased niche partitioning among the species in the fauna. Such reduction in niche partitioning may be consistent with the hypothesis that greater seasonality limits resources and therefore supports fewer taxa in a given niche at higher latitudes [15,18]. Both GM and FK have similar annual mean productivity (1500 mg C/m^2^/day compared to 1600, Table 1), but productivity is much more seasonal in GM as seen through a higher range of annual values (3750 mg C/m^2^/day compared to 2300, Table 1).

### Ornamentation, body size, and predation intensity

Maximum shell size and ornamentation both generally decrease from FK to GM with a stable minimum on both variables. However, in most shared taxonomic and functional groups, the minimum and maximum values stay constant, indicating that fauna-level differences in these attributes are derived from the loss of tropical clades, particularly those with strong defensive ornamentation—i.e. there is taxon sorting with latitude rather than directional shifts within latitudinally persistent clades and functional groups. Further, the highest ornamentation values recorded exclusively in FK represent spines projecting from the shell surface. These spines are hypothesized to be effective deterrents against crushing and peeling predators ([16–17,50], but see [51]). Fish and crustaceans that employ these methods of predation occur throughout the geographic range of this study, but predation intensity is hypothesized to be higher in the tropics [16–17,50,52]. Thus, the presence of highly ornamented and large-bodied species in FK and their absence in GM is consistent with a latitudinal pattern in defense morphology that has also been observed in snails and in some other aspects of bivalve shell form [17,53].

### Proportional shell volume and the cost of calcification

The bivalve shell consists of calcium carbonate and its organic matrix, which are thought to be governed by a trade-off between mechanical needs (e.g. predation defense) and metabolic costs (e.g. resources otherwise necessary for life-maintenance and reproduction). The relation of shell to the total volume of the animal shows no significant change from FK to GM. However, thicker shells and larger ranges of proportional shell volume in GM are inconsistent with the hypothesis that growing and maintaining thicker carbonate shells is costlier at higher latitudes due to either decreased nutrient availability or the under-saturation of carbonate in the ocean [54–55]. Green et al. [32] find that bivalve mortality and dissolution occurs at an aragonite saturation state of ~0.3 Ω_arag._ Aragonite saturation states for FK and GM are 3.9 and 1.5 Ω_arag_ respectively on average, with a minimum of 3.7 and ~1.2 in winter, and 4.1 and 2.5 Ω_arag_ during the warmest months when shell growth is most likely to occur [30–31]. Despite this striking regional difference in saturation states, Ω_arag_ in the study areas does not approach values that would be likely to induce dissolution of shell material, and we see no morphological evidence for “cost” of calcification as a driver of the bivalve LDG. Instead, shell thickness appears to be more closely linked to phylogenetic and/or ecological factors than to the abiotic environment along this particular western ocean boundary, consistent with experimental analyses [56].

## Conclusions

We find that the overall strongest control on regional morphological diversity with latitude is biogeographic filtering of entire families and functional groups, rather than extrinsic factors driving consistent morphological responses within the subgroups—that is, latitudinal sorting of clades and functional groups, rather than morphological transformations within them. The distributions and types of shell form in both regions suggest that high-latitude seasonality in resources and low-latitude predation pressure have relatively stronger effects than do calcification costs. As with many macroevolutionary patterns [8], the principal morphological shifts are not simply directional, as might be hypothesized given the supposed force of the drivers, but represent a shift in trait variance mostly driven by higher-level, clade-sorting processes—thin, plain shells are everywhere, but thick, spiked shells are restricted to the tropics along this coastline.

## Supporting information

S1 Table Specimens included in dataset. Metadata and taxonomic information for scanned specimens and species that are missing from the dataset.
(PDF)

S2 Table Results of statistical tests on MST distances and disparities. Tabulated results for Kolmogorov-Smirnov (MST distances) and Wilcoxon tests (disparities) between faunas and subgroups.
(PDF)

S1 Fig Intraspecific variation. Comparison of 65 FK or GM species with more than 2 individuals scanned (for a wider, as-yet unpublished analysis of bivalve morphology–this dataset includes the FK or GM specimens of these species, plus additional specimens from localities outside of FK or GM which are not included in the main analysis of the paper) for the variables studied in this analysis. Panels A, B, C show species ranges for the three univariate traits. Panels D, E, show the bivariate plots of PCs 1+2, and 2+3 respectively, with the total FK-GM dataset plotted in grey behind the intraspecific variation dataset. Note that most species occupy narrow ranges of values for these traits compared to the overall range of the dataset.
(PDF)

S2 Fig Family subplots of the shape morphospace. Multipanel figure displaying the same data as in Text Fig 3 but faceted by families in order to clarify within-subgroup patterns.
(PDF)

S3 Fig Functional-group subplots of the shape morphospace. Multipanel figure displaying the same data as in Text Fig 3 but faceted by functional groups in order to clarify within-subgroup patterns.
(PDF)

S4 Fig Observed and resampled values for shape space disparities, MST distances, and total morphospace occupation. Faunal disparities, median minimum spanning tree distances, and total morphospace occupation for the whole faunas. Red points are observed values. Black points are the means and black lines are the 95% confidence intervals on the means of 1000 resampled sets.
(PDF)

S5 Fig Observed and resampled values for shape space disparities, MST distances, and total morphospace occupation for families. Faunal disparities, median minimum spanning tree distances, and total morphospace occupation for the families that are shared between regions. Red points are observed values. Black points are means and black lines are 95% confidence intervals on the means of 1000 resampled sets.
(PDF)

S6 Fig Observed and resampled values for shape space disparities, MST distances, and total morphospace occupation for functional groups. Faunal disparities, median minimum spanning tree distances, and total morphospace occupation for the functional groups that are shared between regions. Red points are observed values. Black points are means and black lines are 95% confidence intervals on the means of 1000 resampled sets.
(PDF)

S7 Fig Comparisons of all morphological and shape variables for the twelve species shared between regions. Blue points are GM specimens and orange points are FK specimens. Conspecific individuals are joined with grey lines (note that some specimens are represented by two valves in at least one region, hence grey lines occasionally join points within a region as well as between regions). Note that grey lines cross between regions, suggesting that there is no overarching latitudinal change within species.
(PDF)

S8 Fig Total morphological range as the range of PC scores for each region along each PC axis. (a) The range of scores for each PC axis declines for both the Florida Keys and Gulf of Maine, but the Florida Keys consistently occupies greater range along each PC axis. (b) The cumulative sum of range values for each axis show that the Florida Keys occupies almost double the total morphospace of the Gulf of Maine across 23 PCs (FK = 4627, GM = 2889).
(PDF)

S9 Fig Power analysis of internal shape. (a) Root mean squared error between the true landmark configuration and a landmark configuration reconstructed using the specified subset PCs (i.e. those specified on the x-axis). Each black line represents one specimen. Box plots give the median, inner quartile, and inner 95% of values summarized across specimens at the specified PC. RMSE tends to stabilize by PC 20–25 (94–55% cumulative explained variance). (b) Location of all specimens along PCs 1–2, the specimens used to visualize the power analysis in panel c are highlighted in red. (c) Visualization of reconstructed shapes for specimens plotted in red in panel b. Species with greater shape complexities such as the posterior rostrum in *Cardiomya striata* require more PCs to faithfully reconstruct the general shell shape. Holes in the reconstructed meshes result from generating a mesh surface from reconstructed point-cloud and are not real features of the animal.
(PDF)

S10 Fig Comparison of centroid size with measurements of shell volume. (a) shell volume (i.e. volume of body that is carbonate), (b) internal volume (i.e. soft-tissue+water volume), and (c) shell volume + internal volume.
(PDF)

S1 File R code. R code file for use with S2 File data to rerun analyses and replicate plots for this paper and its supporting figures and tables.
(CSV)

S2 File Supplementary Data. Rdata file for use with S1 File code to rerun analyses and produce plots for this paper and its supporting figures and tables.
(R) Click here for additional data file.
