# Supplementary material for: Spatial filters of function and phylogeny determine morphological disparity with latitude
Source: PLoS One. 2019 Aug 29;14(8):e0221490. doi: 10.1371/journal.pone.0221490 (PMC6715166; doi:10.1371/journal.pone.0221490)
Supplement: S1 Table — Metadata and taxonomic information for scanned specimens and species that are missing from the dataset. (PDF) [file pone.0221490.s001.pdf]

Supplementary Data Table 1: Specimens (meshes) used in the analysis of FK versus GM. Note that specimens with both valves scanned are listed twice, once for each mesh, and that on occasion scan parameters may vary slightly between valves of the same specimen. † Conspecific specimens collected elsewhere within the same target climate zone.

Mesh	Coll.	Catalog #	Valve	Family	Genus (Subgenus)	Species	Authority	Reg.	Locality	Lat	Long	Scan Res (µm)
279	FMNHIZ	329820	L	Anomiidae	*Anomia*	*simplex*	d'Orbigny 1853	FK	† Sanibel Island, West Florida, USA	26.43	-82.12	28.20
749	FMNHIZ	208471	L	Anomiidae	*Pododesmus*	*rudis*	(Broderip 1834)	FK	† Sanibel Island, West Florida, USA	26.43	-82.12	55.24
104	FMNHIZ	183598	L	Arcidae	*Acar*	*domingensis*	(Lamarck 1819)	FK	Missouri Key, Florida Keys, USA	24.68	-81.24	35.33
97	FMNHIZ	183610	L	Arcidae	*Anadara*	*notabilis*	(Röding 1798)	FK	Key West, Florida Keys, USA	24.55	-81.78	35.33
732	FMNHIZ	150205	L	Arcidae	*Anadara (Cunearca)*	*brasiliana*	(Lamarck 1819)	FK	† Amelia Island, East Florida, USA	30.62	-81.44	78.93
1055	USNM	535689	L	Arcidae	*Anadara (Diluvarca)*	*secernenda*	(Lamy 1907)	FK	Florida Keys, USA	24.76	-80.98	33.47
740	FMNHIZ	12227	L	Arcidae	*Anadara (Scapharca)*	*secticostata*	(Reeve 1844)	FK	† Sanibel Island, West Florida, USA	26.43	-82.12	55.36
778	FMNHIZ	183588	L	Arcidae	*Anadara (Scapharca)*	*transversa*	(Say 1822)	FK	† Bradenton Beach, West Florida, USA	27.47	-82.70	41.30
102	FMNHIZ	166532	L	Arcidae	*Arca*	*imbricata*	Bruguière 1789	FK	Key Vaca, Florida Keys, USA	24.76	-80.96	35.33
103	FMNHIZ	183408	L	Arcidae	*Arca*	*zebra*	(Swainson 1833)	FK	Bonefish Key, Florida Keys, USA	24.73	-81.03	35.33
1236	USNM	501759	R	Arcidae	*Asperarca*	*sagrinata*	(Dall 1886)	FK	† Barbados	13.23	-59.53	29.77
111	FMNHIZ	166530	L	Arcidae	*Barbatia*	*cancellaria*	(Lamarck 1819)	FK	Key Vaca, Florida Keys, USA	24.76	-80.96	36.58
91	FMNHIZ	155511	R	Arcidae	*Barbatia (Cucullaearca)*	*candida*	(Helbling 1799)	FK	Key Vaca, Florida Keys, USA	24.76	-80.96	22.08
95	FMNHIZ	194526	L	Arcidae	*Bathyarca*	*glomerula*	(Dall 1881)	FK	SE of Dry Tortugas, Florida Keys, USA	24.63	-82.87	21.17

Mesh	Coll.	Catalog #	Valve	Family	Genus (Subgenus)	Species	Authority	Reg.	Locality	Lat	Long	Scan Res (μm)
101	FMNHIZ	183739	L	Arcidae	*Fugleria*	*tenera*	(C.B. Adams 1845)	FK	Missouri Key, Florida Keys, USA	24.68	-81.24	35.33
98	FMNHIZ	183609	L	Arcidae	*Lunarca*	*ovalis*	(Bruguière 1789)	FK	Bonefish Key, Florida Keys, USA	24.73	-81.03	35.33
798	FMNHIZ	113964	L	Arcidae	*Potiarca[i]*	*chemnitzii*	(Philippi 1851)	FK	† Gulf of Uraba, Colombia	8.34	-76.94	36.30
882	FMNHIZ	208654	L	Astartidae	*Astarte*	*concha*	Huber 2010	FK	Florida Keys, USA	24.76	-80.98	14.07
1237	USNM	330487	L	Astartidae	*Astarte*	*smithii*	Dall 1886	FK	Off Carysfort, Florida Keys, USA	25.22	-80.21	29.77
1087	USNM	765264	R	Astartidae	*Astarte*	*subaequilatera*	Sowerby II, 1854	FK	† E of Fort Lauderdale, East Florida, USA	26.12	-80.10	37.60
198	FMNHIZ	311592	L	Cardiidae	*Acrosterigma*	*magnum*	(Linnaeus 1758)	FK	"The Horseshoe" reef off Marathon, Florida Keys, USA	24.65	-81.26	59.43
109	FMNHIZ	280820	L	Cardiidae	*Americardia*	*guppyi*	(Thiele 1910)	FK	† NE of Nassau, New Providence Island, Bahamas	25.07	-77.28	36.58
118	FMNHIZ	325902	L	Cardiidae	*Americardia*	*media*	(Linnaeus 1758)	FK	† Las Salinas, Dominican Republic	18.25	-71.09	36.58
6	FMNHIZ	176778	L	Cardiidae	*Dallocardia*	*muricata*	(Linnaeus 1758)	FK	Little Duck Key, Florida Keys, USA	24.68	-81.23	84.12
210	FMNHIZ	176765	L	Cardiidae	*Dinocardium*	*robustum*	(Lightfoot 1786)	FK	Fort Pierce, East Florida, USA	27.43	-80.33	104.24
260	FMNHIZ	167521	L	Cardiidae	*Laevicardium*	*mortoni*	(Conrad 1831)	FK	† Punta Rassa, West Florida, USA	26.50	-82.01	35.70
187	FMNHIZ	164110	L	Cardiidae	*Laevicardium*	*pictum*	(Ravenel 1861)	FK	Florida Keys, USA	24.76	-80.98	38.98
1182	USNM	93921	L	Cardiidae	*Laevicardium*	*pictum*	(Ravenel 1861)	FK	† Gulf of Mexico, USA	25.70	-90.03	32.97

Mesh	Coll.	Catalog #	Valve	Family	Genus (Subgenus)	Species	Authority	Reg.	Locality	Lat	Long	Scan Res (µm)
306	FMNHIZ	176769	L	Cardiidae	*Laevicardium*	*pristis*	(Bory de Saint-Vincent 1827)	FK	† Treasure Island, West Florida, USA	27.77	-82.78	63.16
7	FMNHIZ	227405	L	Cardiidae	*Laevicardium*	*serratum*	(Linnaeus 1758)	FK	Marathon, Florida Keys, USA	24.73	-81.03	84.12
281	FMNHIZ	77937	L	Cardiidae	*Microcardium*	*tinctum*	(Dall 1881)	FK	† Egmont Key, Tampa Bay, West Florida, USA	27.59	-82.76	28.20
23	FMNHIZ	182759	L	Cardiidae	*Papyridea*	*lata*	(Born 1778)	FK	Missouri Key, Florida Keys, USA	24.68	-81.24	84.12
285	FMNHIZ	189372	L	Cardiidae	*Papyridea*	*semisulcata*	(Gray 1825)	FK	† NE of Nassau, New Providence Island, Bahamas	25.07	-77.28	28.20
25	FMNHIZ	301980	L	Cardiidae	*Papyridea*	*soleniformis*	(Bruguière 1789)	FK	Elbow Reef, Crawl Key, Florida Keys, USA	24.75	-80.98	84.12
304	FMNHIZ	12721	L	Cardiidae	*Serripes*	*groenlandicus*	(Mohr 1786)	FK	† Anatalak Bay, Nain Labrador	56.58	-61.58	63.16
26	FMNHIZ	176760	L	Cardiidae	*Trachycardium*	*egmontianum*	(Shuttleworth 1856)	FK	Little Duck Key, Florida Keys, USA	24.68	-81.23	84.12
223	FMNHIZ	185745	L	Cardiidae	*Trigoniocardia*	*antillarum*	(d'Orbigny 1853)	FK	† St Thomas, Virgin Islands	18.35	-64.90	27.63
73	FMNHIZ	311591	L	Carditidae	*Cardites*	*floridanus*	(Conrad 1838)	FK	"The Horseshoe" reef off Marathon, Florida Keys, USA	24.65	-81.26	34.28
492	FMNHIZ	295515	R	Carditidae	*Carditopsis*	*smithii*	(Dall 1896)	FK	Geiger Key, Florida Keys, USA	24.58	-81.66	21.82
498	FMNHIZ	197561	L	Carditidae	*Glans*	*dominguensis*	(d'Orbigny 1853)	FK	SE of Dry Tortugas, Florida Keys, USA	24.63	-82.87	21.82
286	FMNHIZ	208336	L	Carditidae	*Pleuromeris*	*tridentata*	(Say 1826)	FK	† Sanibel Island, West Florida, USA	26.43	-82.12	28.20

Mesh	Coll.	Catalog #	Valve	Family	Genus (Subgenus)	Species	Authority	Reg.	Locality	Lat	Long	Scan Res (µm)
331	FMNHIZ	208337	R	Carditidae	*Pteromeris*	*perplana*	(Conrad 1841)	FK	† Sanibel Island, West Florida, USA	26.43	-82.12	14.68
22	FMNHIZ	163680	L	Chamidae	*Arcinella*	*cornuta*	Conrad 1866	FK	Florida Keys, USA	24.76	-80.98	84.12
166	FMNHIZ	288725	L	Chamidae	*Chama*	*congregata*	Conrad 1833	FK	Missouri Key, Florida Keys, USA	24.68	-81.24	75.79
167	FMNHIZ	288725	R	Chamidae	*Chama*	*congregata*	Conrad 1833	FK	Missouri Key, Florida Keys, USA	24.68	-81.24	75.79
1095	USNM	599356	L	Chamidae	*Chama*	*florida*	Lamarck 1819	FK	Key West, Florida Keys, USA	24.55	-81.78	30.50
1279	USNM	445778	L	Chamidae	*Chama*	*lactuca*	Dall 1886	FK	Sand Key, Florida Keys, USA	24.46	-81.88	33.44
39	FMNHIZ	227408	L	Chamidae	*Chama*	*macerophylla*	Gmelin 1791	FK	Missouri Key, Florida Keys, USA	24.68	-81.24	78.93
44	FMNHIZ	227408	R	Chamidae	*Chama*	*macerophylla*	Gmelin 1791	FK	Missouri Key, Florida Keys, USA	24.68	-81.24	78.93
1281	USNM	53591	L	Chamidae	*Chama*	*sarda*	Reeve 1847	FK	† Egmont Key, Tampa Bay, West Florida, USA	27.59	-82.76	33.44
13	FMNHIZ	183141	R	Chamidae	*Chama*	*sinuosa*	Broderip 1835	FK	Bonefish Key, Florida Keys, USA	24.73	-81.03	84.12
14	FMNHIZ	183141	L	Chamidae	*Chama*	*sinuosa*	Broderip 1835	FK	Bonefish Key, Florida Keys, USA	24.73	-81.03	84.12
708	FMNHIZ	183211	R	Chamidae	*Pseudochama*	*cristella*	(Lamarck 1819)	FK	† Miami Causeway, Miami-Dade County, East Florida, USA	25.79	-80.18	59.36
712	FMNHIZ	183211	L	Chamidae	*Pseudochama*	*cristella*	(Lamarck 1819)	FK	† Miami Causeway, Miami-Dade County, East Florida, USA	25.79	-80.18	59.36
1234	USNM	449654	L	Corbulidae	*Caryocorbula*	*chittyana*	(C.B. Adams 1852)	FK	Dry Tortugas, Florida Keys, USA	24.63	-82.87	29.77

Mesh	Coll.	Catalog #	Valve	Family	Genus (Subgenus)	Species	Authority	Reg.	Locality	Lat	Long	Scan Res (µm)
352	FMNHIZ	306064	R	Corbulidae	*Caryocorbula*	*cymella*	(Dall 1881)	FK	Looe Key, Florida Keys, USA	24.66	-81.41	15.79
342	FMNHIZ	329831	L	Corbulidae	*Caryocorbula*	*dietziana*	(C.B. Adams 1852)	FK	† Sanibel Island, West Florida, USA	26.43	-82.12	14.68
1450	USNM	449934	L	Corbulidae	*Caryocorbula*	*swiftiana*	(C.B. Adams 1852)	FK	† Off Fowey Rocks Lighthouse, Key Biscayne, East Florida, USA	25.59	-80.10	35.86
337	FMNHIZ	306076	L	Corbulidae	*Juliacorbula*	*aequivalvis*	(Philippi 1836)	FK	Looe Key, Florida Keys, USA	24.66	-81.41	14.68
359	FMNHIZ	132402	R	Corbulidae	*Varicorbula*	*philippii*	(Smith 1885)	FK	† Destin, Northwest Florida, USA	30.39	-86.50	15.79
513	FMNHIZ	333141	R	Corbulidae	*Vokesula [ii]*	*limatula*	(Conrad 1846)	FK	Halfmoon Shoal, Florida Keys, USA	24.56	-82.21	30.78
494	FMNHIZ	311562	L	Crassatellidae	*Crassinella*	*dupliniana*	(Dall 1903)	FK	Halfmoon Shoal, Florida Keys, USA	24.56	-82.21	21.82
353	FMNHIZ	306072	R	Crassatellidae	*Crassinella*	*lunulata*	(Conrad 1834)	FK	S of Bahia Honda Key, Florida Keys, USA	24.66	-81.26	15.79
321	FMNHIZ	194518	R	Crassatellidae	*Crassinella*	*martinicensis*	(d'Orbigny 1853)	FK	SE of Dry Tortugas, Florida Keys, USA	24.63	-82.87	7.86
19	FMNHIZ	302046	L	Crassatellidae	*Kalolophus*	*speciosus*	(A. Adams 1854)	FK	Dry Tortugas, Florida Keys, USA	24.63	-82.87	84.12
499	FMNHIZ	26385	L	Cuspidariidae	*Cardiomya*	*costellata*	(Deshayes 1833)	FK	† Lake Worth, East Florida, USA	26.62	-80.04	21.82
1243	USNM	599334	L	Cuspidariidae	*Cardiomya*	*ornatissima*	(d'Orbigny 1853)	FK	† Destin, Northwest Florida, USA	30.39	-86.50	29.77
333	FMNHIZ	311572	R	Cuspidariidae	*Cardiomya*	*perrostrata*	(Dall 1881)	FK	Key Colony Beach, Florida Keys, USA	24.72	-81.02	14.68
1240	USNM	460732	L	Cuspidariidae	*Cardiomya*	*striata*	Jeffreys 1876	FK	Sand Key, Florida Keys, USA	24.46	-81.88	29.77

Mesh	Coll.	Catalog #	Valve	Family	Genus (Subgenus)	Species	Authority	Reg.	Locality	Lat	Long	Scan Res (µm)
1313	USNM	448494	L	Cuspidariidae	*Cuspidaria*	*obesa*	(Lovén 1846)	FK	Sand Key, Florida Keys, USA	24.46	-81.88	32.80
1272	USNM	811161	L	Cuspidariidae	*Cuspidaria*	*rostrata*	(Spengler 1793)	FK	† Paraguaná Peninsula, Venezuela	11.60	-70.01	29.77
2517	USNM	93962	R	Cuspidariidae	*Myonera*	*lamellifera*	Dall 1881	FK	† Cedar Keys, Florida, USA	29.15	-83.04	32.66
1204	USNM	448488	L	Cuspidariidae	*Plectodon*	*granulatus*	(Dall 1881)	FK	Sand Key, Florida Keys, USA	24.46	-81.88	31.09
1252	USNM	421695	L	Dimyidae	*Dimya*	*tigrina*	Bayer 1971	FK	Dry Tortugas, Florida Keys, USA	24.63	-82.87	29.77
110	FMNHIZ	202068	L	Donacidae	*Donax (Paraserrula)*	*variabilis*	(Say 1822)	FK	Matecumbe Key, Florida Keys, USA	24.86	-80.71	36.58
771	FMNHIZ	23505	L	Donacidae	*Iphigenia*	*brasiliensis*	(Lamarck 1818)	FK	† Jupiter Inlet, East Florida, USA	26.94	-80.07	50.89
52	FMNHIZ	189226	L	Gastrochaenidae	*Lamychaena*	*hians*	(Gmelin 1791)	FK	Bonefish Key, Florida Keys, USA	24.73	-81.03	78.93
242	FMNHIZ	189226	R	Gastrochaenidae	*Lamychaena*	*hians*	(Gmelin 1791)	FK	Bonefish Key, Florida Keys, USA	24.73	-81.03	78.93
68	FMNHIZ	188359	L	Gastrochaenidae	*Spengleria*	*rostrata*	(Spengler 1783)	FK	Molasses Key, Florida Keys, USA	24.69	-81.19	34.28
130	FMNHIZ	188359	R	Gastrochaenidae	*Spengleria*	*rostrata*	(Spengler 1783)	FK	Molasses Key, Florida Keys, USA	24.69	-81.19	34.28
29	FMNHIZ	301536	L	Glycymerididae	*Glycymeris*	*decussata*	(Linnaeus 1758)	FK	Sombrero Reef, Florida Keys, USA	24.70	-81.08	84.12
117	FMNHIZ	306123	L	Glycymerididae	*Glycymeris*	*spectralis*	Nicol 1952	FK	Halfmoon Shoal, Florida Keys, USA	24.56	-82.21	36.58
706	FMNHIZ	206064	L	Glycymerididae	*Glycymeris*	*undata*	(Linnaeus 1758)	FK	† Puerto La Cruz, Venezuela	10.22	-64.65	59.36
179	FMNHIZ	150854	L	Glycymerididae	*Tucetona*	*pectinata*	(Gmelin 1791)	FK	Bonefish Key, Florida Keys, USA	24.73	-81.03	38.98
614	FMNHIZ	302056	R	Gryphaeidae	*Hyotissa*	*mcgintyi*	(Harry 1985)	FK	† Alcobaça, State of Bahia, Brazil	-17.55	39.19	98.70

Mesh	Coll.	Catalog #	Valve	Family	Genus (Subgenus)	Species	Authority	Reg.	Locality	Lat	Long	Scan Res (µm)
636	FMNHIZ	302056	L	Gryphaeidae	*Hyotissa*	*mcgintyi*	(Harry 1985)	FK	† Alcobaça, State of Bahia, Brazil	-17.55	39.19	77.94
1081	USNM	516571	L	Gryphaeidae	*Neopycnodonte*	*cochlear*	(Poli 1795)	FK	SE of Dry Tortugas, Florida Keys, USA	24.63	-82.87	37.60
79	FMNHIZ	194525	R	Hiatellidae	*Hiatella*	*arctica*	(Linnaeus 1767)	FK	SE of Dry Tortugas, Florida Keys, USA	24.63	-82.87	34.28
80	FMNHIZ	194525	L	Hiatellidae	*Hiatella*	*arctica*	(Linnaeus 1767)	FK	SE of Dry Tortugas, Florida Keys, USA	24.63	-82.87	34.28
57	FMNHIZ	183223	R	Isognomonidae	*Isognomon*	*alatus*	(Gmelin 1791)	FK	Key Vaca, Florida Keys, USA	24.76	-80.96	87.39
162	FMNHIZ	183227	L	Isognomonidae	*Isognomon*	*bicolor*	(C.B. Adams 1845)	FK	Missouri Key, Florida Keys, USA	24.68	-81.24	75.79
163	FMNHIZ	183227	R	Isognomonidae	*Isognomon*	*bicolor*	(C.B. Adams 1845)	FK	Missouri Key, Florida Keys, USA	24.68	-81.24	75.79
56	FMNHIZ	183228	L	Isognomonidae	*Isognomon*	*radiatus*	(Anton 1839)	FK	Missouri Key, Florida Keys, USA	24.68	-81.24	87.39
59	FMNHIZ	183228	R	Isognomonidae	*Isognomon*	*radiatus*	(Anton 1839)	FK	Missouri Key, Florida Keys, USA	24.68	-81.24	87.39
2532	USNM	429390	R	Limidae	*Ctenoides*	*miamiensis*	Mikkelsen & Bieler 2003	FK	† Dominican Republic, Samana Bay	19.15	-69.15	32.66
45	FMNHIZ	182929	L	Limidae	*Ctenoides*	*mitis*	(Lamarck 1807)	FK	Key West, Florida Keys, USA	24.55	-81.78	78.93
1426	USNM	502747	L	Limidae	*Ctenoides*	*planulatus*	(Dall 1886)	FK	† Lazaretto, Barbados	13.14	-59.64	30.91
1521	USNM	458282	R	Limidae	*Ctenoides*	*sanctipauli*	Stuardo 1982	FK	Sand Key, Florida Keys, USA	24.46	-81.88	51.58
106	FMNHIZ	182936	L	Limidae	*Ctenoides*	*scaber*	(Born 1778)	FK	Pigeon Key, Florida Keys, USA	24.70	-81.16	35.33
1247	USNM	94019	L	Limidae	*Divarilima*	*albicoma*	(Dall 1886)	FK	Key West, Florida Keys, USA	24.55	-81.78	29.77

Mesh	Coll.	Catalog #	Valve	Family	Genus (Subgenus)	Species	Authority	Reg.	Locality	Lat	Long	Scan Res (µm)
99	FMNHIZ	182935	L	Limidae	*Lima*	*caribaea*	d'Orbigny 1853	FK	Missouri Key, Florida Keys, USA	24.68	-81.24	35.33
1274	USNM	61742	R	Limidae	*Limaria*	*locklini*	(McGinty 1955)	FK	† Florida, USA	28.06	-81.58	29.77
213	FMNHIZ	288811	L	Limidae	*Limaria*	*pellucida*	(C.B. Adams 1846)	FK	Florida Keys, USA	24.76	-80.98	27.63
1241	USNM	458298	L	Limidae	*Limatula*	*setifera*	Dall 1886	FK	† Off Fowey Rocks Lighthouse, Key Biscayne, East Florida, USA	25.59	-80.10	29.77
1304	USNM	458309	L	Limidae	*Limea (Gemellima)*	*bronniana*	(Dall 1886)	FK	† Off Fowey Rocks Lighthouse, Key Biscayne, East Florida, USA	25.59	-80.10	20.71
227	FMNHIZ	175808	L	Limopsidae	*Limopsis*	*aurita*	(Brocchi 1814)	FK	SE of Dry Tortugas, Florida Keys, USA	24.63	-82.87	27.63
1210	USNM	93571	L	Limopsidae	*Limopsis*	*sulcata*	Verrill & Bush 1898	FK	† Cape Florida, Key Biscayne, East Florida, USA	25.67	-80.16	31.09
859	FMNHIZ	187483	L	Limopsidae	*Limopsis (Limopsis)*	*minuta*	(Philippi 1836)	FK	† Egmont Key, Tampa Bay, West Florida, USA	27.59	-82.76	29.30
32	FMNHIZ	301628	L	Lucinidae	*Anodontia*	*alba*	Link 1807	FK	Molasses Key, Florida Keys, USA	24.69	-81.19	84.12
219	FMNHIZ	311577	L	Lucinidae	*Callucina*	*keenae*	(Chavan 1971)	FK	Long Key, Florida Keys, USA	24.82	-80.82	27.63
503	FMNHIZ	330944	R	Lucinidae	*Cavilinga*	*blanda*	(Dall, in Dall & Simpson 1901)	FK	Sombrero Reef, Florida Keys, USA	24.70	-81.08	30.78
860	FMNHIZ	13881	L	Lucinidae	*Clathrolucina*	*costata*	(d'Orbigny 1845)	FK	† Pontevedra Beach, East Florida, USA	30.24	-81.38	29.30
46	FMNHIZ	176528	L	Lucinidae	*Codakia*	*orbicularis*	(Linnaeus 1758)	FK	Little Duck Key, Florida Keys, USA	24.68	-81.23	78.93

Mesh	Coll.	Catalog #	Valve	Family	Genus (Subgenus)	Species	Authority	Reg.	Locality	Lat	Long	Scan Res (µm)
1201	USNM	272727	L	Lucinidae	*Ctena*	*orbiculata*	(Montagu 1808)	FK	Boca Grande Key, Florida Keys, USA	24.53	-82.00	31.09
185	FMNHIZ	94727	L	Lucinidae	*Divalinga*	*quadrisulcata*	(d'Orbigny 1845)	FK	Grassy Key, Florida Keys, USA	24.76	-80.95	38.98
89	FMNHIZ	177584	L	Lucinidae	*Divaricella*	*dentata*	(Wood 1815)	FK	Bahia Honda Key, Florida Keys, USA	24.67	-81.26	22.08
2619	UFIZ	398487	L	Lucinidae	*Eulopia*	*sagrinata*	(Dall 1886)	FK	† Biscayne Bay, Cape Florida, USA	25.83	-80.16	23.6
10	FMNHIZ	176532	L	Lucinidae	*Lucina*	*pensylvanica*	(Linnaeus 1758)	FK	Little Duck Key, Florida Keys, USA	24.68	-81.23	84.12
1206	USNM	446328	L	Lucinidae	*Lucinisca*	*muricata*	(Spengler 1798)	FK	† Cabanas Harbor, Cuba	22.98	-82.92	31.09
93	FMNHIZ	311596	L	Lucinidae	*Lucinisca*	*nassula*	(Conrad 1846)	FK	Rabbit Key, Florida Keys, USA	25.75	-81.38	21.17
121	FMNHIZ	306143	L	Lucinidae	*Lucinoma*	*filosa*	(Stimpson 1851)	FK	Dry Tortugas, Florida Keys, USA	24.63	-82.87	36.58
512	FMNHIZ	313652	L	Lucinidae	*Parvilucina*	*crenella*	(Dall 1901)	FK	Garden Key, Dry Tortugas, Florida Keys, USA	24.63	-82.87	30.78
510	FMNHIZ	337539	L	Lucinidae	*Parvilucina*	*pectinella*	(C.B. Adams 1852)	FK	Plantation Key, Florida Keys, USA	24.99	-80.55	30.78
725	FMNHIZ	29938	L	Lucinidae	*Pegophysema*	*schrammi*	(Crosse 1876)	FK	† St George's, Bermuda	32.38	-64.68	78.53
15	FMNHIZ	288709	L	Lucinidae	*Phacoides*	*pectinatus*	(Gmelin 1791)	FK	Fiesta Key, Florida Keys, USA	24.84	-80.79	84.12
354	FMNHIZ	306087	L	Lucinidae	*Pleurolucina*	*leucocyma*	(Dall 1886)	FK	Key Colony Beach, Florida Keys, USA	24.72	-81.02	15.79
335	FMNHIZ	306086	L	Lucinidae	*Pleurolucina*	*sombrerensis*	(Dall 1886)	FK	Key Colony Beach, Florida Keys, USA	24.72	-81.02	14.68
336	FMNHIZ	306088	R	Lucinidae	*Radiolucina*	*amianta*	(Dall 1901)	FK	Ramrod Key, Florida Keys, USA	24.66	-81.41	14.68
826	FMNHIZ	59608	L	Lucinidae	*Stewartia*	*floridana*	(Conrad 1833)	FK	† Florida, USA	28.06	-81.58	32.81

Mesh	Coll.	Catalog #	Valve	Family	Genus (Subgenus)	Species	Authority	Reg.	Locality	Lat	Long	Scan Res (µm)
215	FMNHIZ	227454	L	Lyonsiidae	*Entodesma*	*brasiliense*	("Couthuoy" Gould 1850)	FK	Missouri Key, Florida Keys, USA	24.68	-81.24	27.63
858	FMNHIZ	148368	L	Lyonsiidae	*Lyonsia*	*floridana*	Conrad 1849	FK	† Florida, USA	28.06	-81.58	29.30
713	FMNHIZ	208398	L	Mactridae	*Anatina*	*anatina*	(Spengler 1802)	FK	† Sanibel Island, West Florida, USA	26.43	-82.12	59.36
124	FMNHIZ	183845	L	Mactridae	*Mactrotoma*	*fragilis*	(Gmelin 1791)	FK	Little Duck Key, Florida Keys, USA	24.68	-81.23	36.58
1297	USNM	592295	L	Mactridae	*Mulinia*	*lateralis*	(Say 1822)	FK	† Punta Gorda, West Florida, USA	26.93	-82.09	30.80
735	FMNHIZ	208390	L	Mactridae	*Raeta*	*plicatella*	Lamarck 1818	FK	† Sanibel Island, West Florida, USA	26.43	-82.12	55.36
711	FMNHIZ	158441	L	Mactridae	*Rangia*	*flexuosa*	(Conrad 1839)	FK	† Clear Lake, Texas, USA	29.56	-95.06	59.36
1136	USNM	457011	R	Malleidae	*Malleus*	*candeanus*	(d'Orbigny 1853)	FK	Dry Tortugas, Florida Keys, USA	24.63	-82.87	41.26
1244	USNM	217824	L	Myidae	*Sphenia*	*fragilis*	(H. & A. Adams 1854)	FK	† Magdalena Bay, California, USA	24.59	-112.00	29.77
1200	USNM	161738	L	Mytilidae	*Amygdalum*	*arborescens*	(Fischer von Waldheim 1807)	FK	† Mayagüez, Puerto Rico	18.21	-67.18	31.09
1316	USNM	64092	R	Mytilidae	*Amygdalum*	*politum*	(Verrill & Smith 1880)	FK	Dry Tortugas, Florida Keys, USA	24.63	-82.87	32.80
1294	USNM	810334	R	Mytilidae	*Amygdalum*	*sagittatum*	(Rehder 1935)	FK	† Jensen Beach, Florida, USA	27.25	-80.21	30.80
819	FMNHIZ	208324	R	Mytilidae	*Arcuatula*	*papyria*	(Conrad 1846)	FK	† Sanibel Island, West Florida, USA	26.43	-82.12	31.92
70	FMNHIZ	183684	L	Mytilidae	*Botula*	*fusca*	(Gmelin 1791)	FK	Key Vaca, Florida Keys, USA	24.76	-80.96	34.28
90	FMNHIZ	188293	L	Mytilidae	*Brachidontes*	*exustus*	(Linnaeus 1758)	FK	Matecumbe Key, Florida Keys, USA	24.86	-80.71	22.08
181	FMNHIZ	227434	L	Mytilidae	*Brachidontes*	*modiolus*	(Linnaeus 1767)	FK	Key West, Florida Keys, USA	24.55	-81.78	38.98

Mesh	Coll.	Catalog #	Valve	Family	Genus (Subgenus)	Species	Authority	Reg.	Locality	Lat	Long	Scan Res (µm)
325	FMNHIZ	306071	L	Mytilidae	*Crenella*	*divaricata*	(Montagu 1808)	FK	Key Colony Beach, Florida Keys, USA	24.72	-81.02	7.863
347	FMNHIZ	311566	R	Mytilidae	*Dacrydium (Quendreda)[iii]*	*hendersoni*	Salas & Gofas 1997	FK	Key Colony Beach, Florida Keys, USA	24.72	-81.02	15.79
30	FMNHIZ	166064	L	Mytilidae	*Geukensia*	*granosissima*	(Sowerby III 1914)	FK	Grassy Key, Florida Keys, USA	24.76	-80.95	84.12
876	FMNHIZ	16430	L	Mytilidae	*Gregariella*	*coralliophaga*	(Gmelin 1791)	FK	† Challenger Bank, Bermuda	32.08	-65.05	29.30
237	FMNHIZ	227439	L	Mytilidae	*Ischadium*	*recurvum*	(Rafinesque 1820)	FK	Key West, Florida Keys, USA	24.55	-81.78	39.54
69	FMNHIZ	183671	L	Mytilidae	*Leiosolenus*	*aristatus*	(Dillwyn 1817)	FK	Missouri Key, Florida Keys, USA	24.68	-81.24	34.28
131	FMNHIZ	183671	R	Mytilidae	*Leiosolenus*	*aristatus*	(Dillwyn 1817)	FK	Missouri Key, Florida Keys, USA	24.68	-81.24	34.28
67	FMNHIZ	183635	L	Mytilidae	*Leiosolenus (Diberus)*	*bisulcatus*	(d'Orbigny 1853)	FK	Bonefish Key, Florida Keys, USA	24.73	-81.03	34.28
243	FMNHIZ	183635	R	Mytilidae	*Leiosolenus (Diberus)*	*bisulcatus*	(d'Orbigny 1853)	FK	Bonefish Key, Florida Keys, USA	24.73	-81.03	34.28
823	FMNHIZ	12212	L	Mytilidae	*Lioberus*	*castanea*	(Say 1822)	FK	† Sanibel Island, West Florida, USA	26.43	-82.12	31.92
47	FMNHIZ	183716	R	Mytilidae	*Lithophaga*	*antillarum*	(d'Orbigny 1853)	FK	Bonefish Key, Florida Keys, USA	24.73	-81.03	78.93
66	FMNHIZ	227440	L	Mytilidae	*Lithophaga*	*nigra*	(d'Orbigny 1853)	FK	Missouri Key, Florida Keys, USA	24.68	-81.24	34.28
33	FMNHIZ	183706	R	Mytilidae	*Modiolus*	*americanus*	(Leach, in Leach & Nodder 1815)	FK	Missouri Key, Florida Keys, USA	24.68	-81.24	84.12
278	FMNHIZ	329833	L	Mytilidae	*Modiolus*	*squamosus*	Beauperthuy 1967	FK	† Sanibel Island, West Florida, USA	26.43	-82.12	28.20
86	FMNHIZ	183678	L	Mytilidae	*Musculus (Modiolarca)*	*lateralis*	(Say 1822)	FK	Matecumbe Key, Florida Keys, USA	24.86	-80.71	34.28

Mesh	Coll.	Catalog #	Valve	Family	Genus (Subgenus)	Species	Authority	Reg.	Locality	Lat	Long	Scan Res (µm)
100	FMNHIZ	94728	L	Noetiidae	*Arcopsis*	*adamsi*	(Dall 1886)	FK	Key West, Florida Keys, USA	24.55	-81.78	35.33
96	FMNHIZ	184106	L	Noetiidae	*Noetia (Eontia)*	*ponderosa*	(Say 1822)	FK	Key West, Florida Keys, USA	24.55	-81.78	35.33
495	FMNHIZ	376277	L	Nucinellidae	*Nucinella*	*adamsii*	(Dall 1898)	FK	Florida Keys, USA	24.76	-80.98	21.82
501	FMNHIZ	376280	L	Nuculanidae	*Propeleda*	*carpenteri*	(Dall 1881)	FK	Florida Keys, USA	24.76	-80.98	21.82
1388	USNM	323936a	L	Nuculanidae	*Propeleda*	*platessa*	(Dall 1890)	FK	Cape San Blas, Northwest Florida, USA	29.66	-85.36	33.39
94	FMNHIZ	195998	R	Nuculanidae	*Saccella*	*acuta*	(Conrad 1831)	FK	SE of Dry Tortugas, Florida Keys, USA	24.63	-82.87	21.17
497	FMNHIZ	317640	R	Nuculanidae	*Saccella*	*concentrica*	(Say 1824)	FK	Florida Keys, USA	24.76	-80.98	21.82
1233	USNM	540716	R	Nuculanidae	*Saccella*	*verrilliana*	(Dall 1886)	FK	† Vero Beach, East Florida, USA	27.65	-80.37	29.77
1312	USNM	1446648	L	Nuculanidae	*Saccella*	*vitrea*	(d'Orbigny in Sagra 1853)	FK	† Ensenada de Cochines, Cuba	22.17	-81.17	20.71
1383	USNM	80029	L	Nuculidae	*Ennucula*	*tenuis*	(Montagu 1808)	FK	† Daytona Beach, East Florida, USA	29.22	-81.00	33.39
339	FMNHIZ	208321	L	Nuculidae	*Nucula*	*proxima*	Say 1822	FK	† Sanibel Island, West Florida, USA	26.43	-82.12	14.68
355	FMNHIZ	306096	L	Nuculidae	*Nucula (Lamellinucula)*	*crenulata*	(A. Adams 1856)	FK	Key Colony Beach, Florida Keys, USA	24.72	-81.02	15.79
731	FMNHIZ	306124	L	Ostreidae	*Crassostrea*	*rhizophorae*	(Guilding 1828)	FK	† Sanibel Island, West Florida, USA	26.43	-82.12	59.70
734	FMNHIZ	306124	R	Ostreidae	*Crassostrea*	*rhizophorae*	(Guilding 1828)	FK	† Sanibel Island, West Florida, USA	26.43	-82.12	55.36
42	FMNHIZ	311607	L	Ostreidae	*Crassostrea*	*virginica*	(Gmelin 1791)	FK	Key Largo, Florida Keys, USA	25.14	-80.40	78.93
43	FMNHIZ	311607	R	Ostreidae	*Crassostrea*	*virginica*	(Gmelin 1791)	FK	Key Largo, Florida Keys, USA	25.14	-80.40	78.93

Mesh	Coll.	Catalog #	Valve	Family	Genus (Subgenus)	Species	Authority	Reg.	Locality	Lat	Long	Scan Res (μm)
182	FMNHIZ	155510	L	Ostreidae	*Dendostrea*	*frons*	(Linnaeus 1758)	FK	Key West, Florida Keys, USA	24.55	-81.78	38.98
183	FMNHIZ	155510	R	Ostreidae	*Dendostrea*	*frons*	(Linnaeus 1758)	FK	Key West, Florida Keys, USA	24.55	-81.78	38.98
765	FMNHIZ	26426	L	Ostreidae	*Ostrea*	*permollis*	Sowerby II 1871	FK	† Destin, Northwest Florida, USA	30.39	-86.50	50.89
768	FMNHIZ	26426	R	Ostreidae	*Ostrea*	*permollis*	Sowerby II 1871	FK	† Destin, Northwest Florida, USA	30.39	-86.50	50.89
138	FMNHIZ	279417	R	Ostreidae	*Ostrea*	*stentina[iv]*	(Payraudeau 1826)	FK	"The Horseshoe" reef off Marathon, Florida Keys, USA	24.65	-81.26	84.12
53	FMNHIZ	198077	R	Ostreidae	*Teskeyostrea*	*weberi*	(Olsson 1951)	FK	Big Pine Key, Florida Keys, USA	24.68	-81.36	78.93
129	FMNHIZ	198077	L	Ostreidae	*Teskeyostrea*	*weberi*	(Olsson 1951)	FK	Big Pine Key, Florida Keys, USA	24.68	-81.36	78.93
1219	USNM	890097	L	Pandoridae	*Pandora (Pandorella)*	*arenosa*	Conrad 1834	FK	† Lake Worth, East Florida, USA	26.62	-80.04	31.09
1203	USNM	444763	L	Pandoridae	*Pandora (Pandorella)*	*bushiana*	Dall 1886	FK	† Bahia Honda, Cuba	22.94	-83.17	31.09
20	FMNHIZ	194550	R	Pectinidae	*Aequipecten*	*glyptus*	(Verrill 1882)	FK	SE of Dry Tortugas, Florida Keys, USA	24.63	-82.87	84.12
135	FMNHIZ	194550	L	Pectinidae	*Aequipecten*	*glyptus*	(Verrill 1882)	FK	SE of Dry Tortugas, Florida Keys, USA	24.63	-82.87	84.12
49	FMNHIZ	202917	L	Pectinidae	*Antillipecten*	*antillarum*	(Recluz 1853)	FK	Florida Keys, USA	24.76	-80.98	78.93
126	FMNHIZ	202917	R	Pectinidae	*Antillipecten*	*antillarum*	(Recluz 1853)	FK	Florida Keys, USA	24.76	-80.98	78.93
3	FMNHIZ	163671	L	Pectinidae	*Argopecten*	*gibbus*	(Linnaeus 1758)	FK	Key West, Florida Keys, USA	24.55	-81.78	84.12
137	FMNHIZ	163671	R	Pectinidae	*Argopecten*	*gibbus*	(Linnaeus 1758)	FK	Key West, Florida Keys, USA	24.55	-81.78	84.12

Mesh	Coll.	Catalog #	Valve	Family	Genus (Subgenus)	Species	Authority	Reg.	Locality	Lat	Long	Scan Res (µm)
1126	USNM	859902	L	Pectinidae	*Argopecten*	*irradians concentricus*	(Say 1822)	FK	Florida Keys, USA	24.76	-80.98	53.95
201	FMNHIZ	173018	R	Pectinidae	*Argopecten*	*irradians irradians*	(Lamarck 1819)	FK	Key West, Florida Keys, USA	24.55	-81.78	59.43
202	FMNHIZ	173018	L	Pectinidae	*Argopecten*	*irradians irradians*	(Lamarck 1819)	FK	Key West, Florida Keys, USA	24.55	-81.78	59.43
87	FMNHIZ	77916	R	Pectinidae	*Argopecten*	*lineolaris*	(Lamarck 1819)	FK	Dry Tortugas, Florida Keys, USA	24.63	-82.87	22.08
88	FMNHIZ	77916	L	Pectinidae	*Argopecten*	*lineolaris*	(Lamarck 1819)	FK	Dry Tortugas, Florida Keys, USA	24.63	-82.87	22.08
71	FMNHIZ	227448	L	Pectinidae	*Argopecten*	*nucleus*	(Born 1778)	FK	Little Duck Key, Florida Keys, USA	24.68	-81.23	34.28
132	FMNHIZ	227448	R	Pectinidae	*Argopecten*	*nucleus*	(Born 1778)	FK	Little Duck Key, Florida Keys, USA	24.68	-81.23	34.28
751	FMNHIZ	183444	R	Pectinidae	*Caribachlamys*	*mildredae*	(Bayer 1941)	FK	† Miami, East Florida, USA	25.76	-80.18	55.24
764	FMNHIZ	183444	L	Pectinidae	*Caribachlamys*	*mildredae*	(Bayer 1941)	FK	† Miami, East Florida, USA	25.76	-80.18	50.89
747	FMNHIZ	183442	R	Pectinidae	*Caribachlamys*	*ornata*	(Lamarck 1819)	FK	† Miami, East Florida, USA	25.76	-80.18	55.24
754	FMNHIZ	183442	L	Pectinidae	*Caribachlamys*	*ornata*	(Lamarck 1819)	FK	† Miami, East Florida, USA	25.76	-80.18	55.24
12	FMNHIZ	183577	R	Pectinidae	*Caribachlamys*	*pellucens*	(Linnaeus 1758)	FK	Garden Key, Dry Tortugas, Florida Keys, USA	24.63	-82.87	84.12
136	FMNHIZ	183577	L	Pectinidae	*Caribachlamys*	*pellucens*	(Linnaeus 1758)	FK	Garden Key, Dry Tortugas, Florida Keys, USA	24.63	-82.87	84.12
159	FMNHIZ	183540	L	Pectinidae	*Caribachlamys*	*sentis*	(Reeve 1853)	FK	Key West, Florida Keys, USA	24.55	-81.78	75.79
160	FMNHIZ	183540	R	Pectinidae	*Caribachlamys*	*sentis*	(Reeve 1853)	FK	Key West, Florida Keys, USA	24.55	-81.78	75.79
827	FMNHIZ	203581	L	Pectinidae	*Cryptopecten*	*phrygium*	(Dall 1886)	FK	† Palm Beach, East Florida, USA	26.71	-80.03	32.81

Mesh	Coll.	Catalog #	Valve	Family	Genus (Subgenus)	Species	Authority	Reg.	Locality	Lat	Long	Scan Res (µm)
833	FMNHIZ	203581	R	Pectinidae	*Cryptopecten*	*phrygium*	(Dall 1886)	FK	† Palm Beach, East Florida, USA	26.71	-80.03	32.81
805	FMNHIZ	183552	R	Pectinidae	*Euvola*	*chazaliei*	(Dautzenberg 1900)	FK	† Palm Beach, East Florida, USA	26.71	-80.03	31.92
830	FMNHIZ	183552	L	Pectinidae	*Euvola*	*chazaliei*	(Dautzenberg 1900)	FK	† Palm Beach, East Florida, USA	26.71	-80.03	32.81
195	FMNHIZ	302047	R	Pectinidae	*Euvola*	*laurentii*	(Gmelin 1791)	FK	Dry Tortugas, Florida Keys, USA	24.63	-82.87	59.43
196	FMNHIZ	302047	L	Pectinidae	*Euvola*	*laurentii*	(Gmelin 1791)	FK	Dry Tortugas, Florida Keys, USA	24.63	-82.87	59.43
24	FMNHIZ	183572	R	Pectinidae	*Euvola*	*raveneli*	(Dall 1898)	FK	Key West, Florida Keys, USA	24.55	-81.78	84.12
28	FMNHIZ	183572	L	Pectinidae	*Euvola*	*raveneli*	(Dall 1898)	FK	Key West, Florida Keys, USA	24.55	-81.78	84.12
40	FMNHIZ	183571	R	Pectinidae	*Euvola*	*ziczac*	(Linnaeus 1758)	FK	Key West, Florida Keys, USA	24.55	-81.78	78.93
41	FMNHIZ	183571	L	Pectinidae	*Euvola*	*ziczac*	(Linnaeus 1758)	FK	Key West, Florida Keys, USA	24.55	-81.78	78.93
1113	USNM	846201	L	Pectinidae	*Laevichlamys*	*multisquamata*	(Dunker 1864)	FK	† Boynton Beach, East Florida, USA	26.55	-80.05	53.95
1114	USNM	846201	R	Pectinidae	*Laevichlamys*	*multisquamata*	(Dunker 1864)	FK	† Boynton Beach, East Florida, USA	26.55	-80.05	53.95
1195	USNM	846221	R	Pectinidae	*Lindapecten[v]*	*exasperatus*	(Sowerby II 1842)	FK	Key Vaca, Florida Keys, USA	24.76	-80.96	32.97
1196	USNM	846221	L	Pectinidae	*Lindapecten*[ii]	*exasperatus*	(Sowerby II 1842)	FK	Key Vaca, Florida Keys, USA	24.76	-80.96	32.97
2	FMNHIZ	183554	L	Pectinidae	*Lindapecten*[ii]	*muscosus*	(Wood 1828)	FK	Key West, Florida Keys, USA	24.55	-81.78	84.12
37	FMNHIZ	302048	L	Pectinidae	*Nodipecten*	*fragosus*	(Conrad 1849)	FK	Dry Tortugas, Florida Keys, USA	24.63	-82.87	78.93

Mesh	Coll.	Catalog #	Valve	Family	Genus (Subgenus)	Species	Authority	Reg.	Locality	Lat	Long	Scan Res (µm)
75	FMNHIZ	170975	R	Pectinidae	*Spathochlamys*	*benedicti*	(Verrill & Bush in Verrill 1897)	FK	SE of Dry Tortugas, Florida Keys, USA	24.63	-82.87	34.28
133	FMNHIZ	170975	L	Pectinidae	*Spathochlamys*	*benedicti*	(Verrill & Bush in Verrill 1897)	FK	SE of Dry Tortugas, Florida Keys, USA	24.63	-82.87	34.28
1370	USNM	608799	R	Periplomatidae	*Periploma*	*inequale*	(C. B. Adams 1842)	FK	† Cocoa Beach, East Florida, USA	28.34	-80.60	30.66
234	FMNHIZ	59670	L	Pharidae	*Ensis*	*megistus*	Pilsbry & McGinty 1943	FK	† St Petersburg, West Florida, USA	27.78	-82.66	39.54
881	FMNHIZ	163615	L	Philobryidae	*Cratis*	*antillensis*	(Dall 1881)	FK	† Gulf of Mexico, USA	25.70	-90.03	14.07
1132	USNM	1409682	L	Pholadidae	*Barnea (Anchomasa)*	*truncata*	(Say 1822)	FK	† Boca Ciega Bay, West Florida, USA	27.79	-82.77	41.26
1149	USNM	818293	L	Pholadidae	*Cyrtopleura (Scobinopholas)*	*costata*	(Linnaeus 1758)	FK	† Sanibel Island, West Florida, USA	26.43	-82.12	70.34
1258	USNM	404233	R	Pholadidae	*Martesia*	*striata*	(Linnaeus 1758)	FK	Stock Island, Key West, Florida Keys, USA	24.57	-81.74	29.77
2223	UFIZ	471653	R	Pholadidae	*Particoma*	*cuneiformis*	(Say 1822)	FK	Key West, Florida Keys, USA	24.55	-81.78	43.04
1150	USNM	407370	L	Pholadidae	*Pholas (Thovana)*	*campechiensis*	(Gmelin 1791)	FK	† St Augustine, East Florida, USA	29.90	-81.32	70.34
62	FMNHIZ	184076	L	Pinnidae	*Atrina*	*rigida*	(Lightfoot 1786)	FK	Little Duck Key, Florida Keys, USA	24.68	-81.23	120.88
61	FMNHIZ	151482	L	Pinnidae	*Atrina (Servatrina)*	*seminuda*	(Lamarck 1819)	FK	† Port Isabel, Texas, USA	26.08	-97.21	120.88
63	FMNHIZ	184062	L	Pinnidae	*Atrina (Servatrina)*	*serrata*	(Sowerby 1825)	FK	Little Duck Key, Florida Keys, USA	24.68	-81.23	120.88
60	FMNHIZ	183249	L	Pinnidae	*Pinna*	*carnea*	Gmelin 1791	FK	Bonefish Key, Florida Keys, USA	24.73	-81.03	120.88

Mesh	Coll.	Catalog #	Valve	Family	Genus (Subgenus)	Species	Authority	Reg.	Locality	Lat	Long	Scan Res (µm)
164	FMNHIZ	197534	R	Plicatulidae	*Plicatula*	*gibbosa*	Lamarck 1801	FK	SE of Dry Tortugas, Florida Keys, USA	24.63	-82.87	75.79
165	FMNHIZ	197534	L	Plicatulidae	*Plicatula*	*gibbosa*	Lamarck 1801	FK	SE of Dry Tortugas, Florida Keys, USA	24.63	-82.87	75.79
214	FMNHIZ	311533	R	Poromyidae	*Poromya*	*granulata*	(Nyst & Westendorp 1839)	FK	Florida Keys, USA	24.76	-80.98	27.63
1401	USNM	667635	L	Poromyidae	*Poromya*	*rostrata*	Rehder 1943	FK	† Campeche, Yucatan, Mexico	19.85	-90.55	33.39
862	FMNHIZ	312496	R	Propeamussiidae	*Cyclopecten*	*thalassinus*	(Dall 1886)	FK	† Gulf of Mexico, USA	25.70	-90.03	29.30
81	FMNHIZ	194514	R	Propeamussiidae	*Parvamussium*	*marmoratum*	(Dall 1881)	FK	SE of Dry Tortugas, Florida Keys, USA	24.63	-82.87	34.28
134	FMNHIZ	194514	L	Propeamussiidae	*Parvamussium*	*marmoratum*	(Dall 1881)	FK	SE of Dry Tortugas, Florida Keys, USA	24.63	-82.87	34.28
211	FMNHIZ	194541	R	Propeamussiidae	*Parvamussium*	*sayanum*	(Dall 1886)	FK	SE of Dry Tortugas, Florida Keys, USA	24.63	-82.87	27.63
350	FMNHIZ	311598	L	Propeamussiidae	*Similipecten*	*nanus*	(Verrill & Bush in Verrill 1897)	FK	Key Colony Beach, Florida Keys, USA	24.72	-81.02	15.79
16	FMNHIZ	151500	L	Psammobiidae	*Asaphis*	*deflorata*	(Linnaeus 1758)	FK	Key West, Florida Keys, USA	24.55	-81.78	84.12
72	FMNHIZ	315754	L	Psammobiidae	*Gari (Dysmea)*	*circe*	(Mörch 1876)	FK	Key Largo, Florida Keys, USA	25.14	-80.40	34.28
65	FMNHIZ	295264	L	Psammobiidae	*Heterodonax*	*bimaculatus*	(Linnaeus 1758)	FK	Florida Keys, USA	24.76	-80.98	34.28
1106	USNM	589922	L	Psammobiidae	*Sanguinolaria*	*sanguinolenta*	(Gmelin 1791)	FK	† Santa Barbara de Samana, Dominican Republic	19.20	-69.33	36.55
58	FMNHIZ	227467	L	Pteriidae	*Pinctada*	*imbricata*	(Roeding 1798)	FK	Missouri Key, Florida Keys, USA	24.68	-81.24	87.39

Mesh	Coll.	Catalog #	Valve	Family	Genus (Subgenus)	Species	Authority	Reg.	Locality	Lat	Long	Scan Res (µm)
235	FMNHIZ	302080	L	Pteriidae	*Pinctada*	*longisquamosa*	(Dunker 1852)	FK	Florida Keys, USA	24.76	-80.98	39.54
27	FMNHIZ	183297	R	Pteriidae	*Pteria*	*colymbus*	(Roeding 1798)	FK	Missouri Key, Florida Keys, USA	24.68	-81.24	84.12
82	FMNHIZ	306066	L	Semelidae	*Abra*	*aequalis*	(Say 1822)	FK	Looe Key, Florida Keys, USA	24.66	-81.41	34.28
834	FMNHIZ	208415	L	Semelidae	*Abra*	*lioica*	(Dall 1881)	FK	† Sanibel Island, West Florida, USA	26.43	-82.12	32.81
1265	USNM	95390	L	Semelidae	*Abra*	*longicallus*	(Scacchi 1835)	FK	† St. Kitts	17.36	-62.76	29.77
872	FMNHIZ	202881	L	Semelidae	*Cumingia*	*antillarum*	A. Adams 1850	FK	† Punta Gorda, West Florida, USA	26.93	-82.09	29.30
83	FMNHIZ	202916	L	Semelidae	*Cumingia*	*vanhyningi*	Rehder 1939	FK	Bonefish Key, Florida Keys, USA	24.73	-81.03	34.28
500	FMNHIZ	315644	L	Semelidae	*Ervilia*	*concentrica*	(Holmes 1860)	FK	† Bear Key, Florida, USA	26.65	-82.10	21.82
1250	USNM	462454	L	Semelidae	*Ervilia*	*nitens*	(Montagu 1806)	FK	Conch Key, Florida Keys, USA	27.84	-82.83	29.77
1301	USNM	271972	R	Semelidae	*Ervilia*	*subcancellata[vi]*	E. A. Smith 1885	FK	Loggerhead Key, Dry Tortugas, Florida Keys, USA	24.63	-82.92	20.71
230	FMNHIZ	177985	L	Semelidae	*Semele*	*bellastriata*	(Conrad 1837)	FK	Missouri Key, Florida Keys, USA	24.68	-81.24	27.63
178	FMNHIZ	177473	L	Semelidae	*Semele*	*proficua*	(Pultney 1799)	FK	Missouri Key, Florida Keys, USA	24.68	-81.24	38.98
271	FMNHIZ	135705	R	Semelidae	*Semele (Amphidesma)*	*purpurascens*	(Gmelin 1791)	FK	† Cedar Keys, West Florida, USA	29.14	-83.04	34.04
1232	USNM	438905	L	Semelidae	*Semelina*	*nuculoides*	(Conrad 1841)	FK	Key West, Florida Keys, USA	24.55	-81.78	29.77
275	FMNHIZ	177600	L	Solecurtidae	*Solecurtus*	*cumingianus*	(Dunker 1861)	FK	† Lake Worth, East Florida, USA	26.62	-80.04	34.04

Mesh	Coll.	Catalog #	Valve	Family	Genus (Subgenus)	Species	Authority	Reg.	Locality	Lat	Long	Scan Res (µm)
769	FMNHIZ	177579	L	Solecurtidae	*Tagelus*	*plebeius*	(Lightfoot 1786)	FK	† Fort Pierce, East Florida, USA	27.44	-80.32	50.89
238	FMNHIZ	177617	L	Solecurtidae	*Tagelus (Mesopleura)*	*divisus*	(Spengler 1794)	FK	† Florida, USA	28.06	-81.58	39.54
1431	USNM	451214	L	Solemyidae	*Solemya (Petrasma)*	*occidentalis*	Deshayes 1857	FK	† Santa Rosa, Cuba	22.40	-80.38	30.91
1305	USNM	447043	L	Spheniopsidae	*Spheniopsis*	*triquetra*	(Verrill & Bush 1898)	FK	Florida Keys, USA	24.76	-80.98	20.71
173	FMNHIZ	177515	L	Spondylidae	*Spondylus*	*americanus*	Hermann 1781	FK	Garden Key, Dry Tortugas, Florida Keys, USA	24.63	-82.87	65.81
174	FMNHIZ	177515	R	Spondylidae	*Spondylus*	*americanus*	Hermann 1781	FK	Garden Key, Dry Tortugas, Florida Keys, USA	24.63	-82.87	65.81
733	FMNHIZ	165823	L	Spondylidae	*Spondylus*	*gussonii*	Costa 1829	FK	† W of Clearwater, West Florida, USA	27.97	82.81	55.36
775	FMNHIZ	165823	R	Spondylidae	*Spondylus*	*gussonii*	Costa 1829	FK	† W of Clearwater, West Florida, USA	27.97	82.81	41.30
193	FMNHIZ	177525	R	Spondylidae	*Spondylus*	*tenuis*	Schreibers 1793	FK	Missouri-Little Duck Bridge, Florida Keys, USA	24.68	-81.24	59.43
194	FMNHIZ	177525	L	Spondylidae	*Spondylus*	*tenuis*	Schreibers 1793	FK	Missouri-Little Duck Bridge, Florida Keys, USA	24.68	-81.24	59.43
357	FMNHIZ	202886	L	Tellinidae	*Acorylus*	*gouldii*	(Hanley 1846)	FK	† Lake Worth, East Florida, USA	26.62	-80.04	15.79
1440	USNM	461886	L	Tellinidae	*Ameritella*	*agilis*	(Stimpson 1857)	FK	Key West, Florida Keys, USA	24.55	-81.78	30.91
1254	USNM	462118	R	Tellinidae	*Ameritella*	*consobrina*	(d'Orbigny in Sagra 1853)	FK	† Fowey Rocks Lighthouse, Key Biscayne,	25.59	-80.10	29.77

Mesh	Coll.	Catalog #	Valve	Family	Genus (Subgenus)	Species	Authority	Reg.	Locality	Lat	Long	Scan Res (µm)
									East Florida, USA			
925	FMNHIZ	84253	L	Tellinidae	*Ameritella*	*mitchelli*	(Dall 1895)	FK	† Lake Pontchartrain, Louisiana, USA	30.20	-90.09	31.00
225	FMNHIZ	306106	R	Tellinidae	*Ameritella*	*probrina*	(Boss 1964)	FK	Florida Keys, USA	24.76	-80.98	27.63
348	FMNHIZ	306114	L	Tellinidae	*Ameritella*	*sybaritica*	(Dall 1881)	FK	Key Colony Beach, Florida Keys, USA	24.72	-81.02	15.79
1231	USNM	61174	L	Tellinidae	*Ameritella*	*texana*	(Dall 1900)	FK	† St Augustine, East Florida, USA	29.90	-81.32	29.77
338	FMNHIZ	101697	L	Tellinidae	*Ameritella*	*versicolor*	(DeKay 1843)	FK	† Crystal Beach, West Florida, USA	28.09	-82.78	14.68
189	FMNHIZ	150179	L	Tellinidae	*Austromacoma*	*constricta*	(Bruguière 1792)	FK	Lossman's Key, Florida Keys, USA	24.76	-80.98	38.98
1248	USNM	461726	L	Tellinidae	*Coanyax*	*americanus*	(Dall 1900)	FK	† Off Fowey Rocks Lighthouse, Key Biscayne, East Florida, USA	25.59	-80.10	29.77
356	FMNHIZ	306067	L	Tellinidae	*Cymatoica*	*hendersoni*	Rehder 1939	FK	Ramrod Key, Florida Keys, USA	24.66	-81.41	15.79
984	FMNHIZ	177939	L	Tellinidae	*Cymatoica*	*orientalis*	(Dall 1890)	FK	† Palm Beach, East Florida, USA	26.71	-80.03	32.70
236	FMNHIZ	151345	L	Tellinidae	*Eurytellina*	*alternata*	(Say 1822)	FK	Florida Keys, USA	24.76	-80.98	39.54
762	FMNHIZ	7114	L	Tellinidae	*Eurytellina*	*angulosa*	(Gmelin 1791)	FK	† Amelia Island, East Florida, USA	30.62	-81.44	50.89
51	FMNHIZ	151457	L	Tellinidae	*Eurytellina*	*lineata*	(Turton 1819)	FK	Key West, Florida Keys, USA	24.55	-81.78	78.93
127	FMNHIZ	151457	R	Tellinidae	*Eurytellina*	*lineata*	(Turton 1819)	FK	Key West, Florida Keys, USA	24.55	-81.78	78.93

Mesh	Coll.	Catalog #	Valve	Family	Genus (Subgenus)	Species	Authority	Reg.	Locality	Lat	Long	Scan Res (µm)
241	FMNHIZ	164969	L	Tellinidae	*Eurytellina*	*nitens*	(C.B.Adams 1845)	FK	Key West, Florida Keys, USA	24.55	-81.78	39.54
757	FMNHIZ	7134	R	Tellinidae	*Eurytellina*	*punicea*	(Born 1778)	FK	† Jamaica	18.22	-77.35	55.24
175	FMNHIZ	177483	L	Tellinidae	*Johnsonella*	*fausta*	(Pultney 1799)	FK	Little Duck Key, Florida Keys, USA	24.68	-81.23	65.81
204	FMNHIZ	311628	R	Tellinidae	*Laciolina*	*laevigata*	(Linnaeus 1758)	FK	Florida Keys, USA	24.76	-80.98	59.43
916	FMNHIZ	29926	L	Tellinidae	*Laciolina*	*magna*	(Spengler 1798)	FK	† Harrington Sound, Bermuda	32.33	-64.72	46.61
206	FMNHIZ	311627	L	Tellinidae	*Leporimetis*	*ephippium*	(Spengler 1798)	FK	Molasses Key, Florida Keys, USA	24.69	-81.19	59.43
1224	USNM	438697	R	Tellinidae	*Macoploma*	*extenuata*	(Dall 1900)	FK	Key West, Florida Keys, USA	24.55	-81.78	29.77
1262	USNM	438687	L	Tellinidae	*Macoploma*	*limula*	(Dall 1895)	FK	† Miami, East Florida, USA	25.76	-80.18	29.77
504	FMNHIZ	177942	L	Tellinidae	*Macoploma*	*tageliformis*	(Dall 1900)	FK	Key West, Florida Keys, USA	24.55	-81.78	30.78
240	FMNHIZ	170986	L	Tellinidae	*Macoploma*	*tenta*	(Say 1834)	FK	SE of Dry Tortugas, Florida Keys, USA	24.63	-82.87	39.54
1336	USNM	381903	R	Tellinidae	*Merisca*	*cristallina*	(Spengler 1798)	FK	† Barcelona, Anzoategui, Venezuela	10.15	-64.71	35.68
217	FMNHIZ	197581	L	Tellinidae	*Phyllodina*	*squamifera*	(Deshayes 1855)	FK	SE of Dry Tortugas, Florida Keys, USA	24.63	-82.87	27.63
1268	USNM	461775	L	Tellinidae	*Phyllodina*	*tenuisculpta*	(G. B. Sowerby II 1868)	FK	Key West, Florida Keys, USA	24.55	-81.78	29.77
233	FMNHIZ	202849	R	Tellinidae	*Psammotreta*	*brevifrons*	(Say 1838)	FK	Missouri Key, Florida Keys, USA	24.68	-81.24	27.63
1414	USNM	429628	L	Tellinidae	*Pseudomacalia*	*antillarum*	(d'Orbigny 1853)	FK	† Samana Bay, Dominican Republic (Johnson-Smithsonian Deep-Sea Exp, station 55)	19.15	-69.14	30.91

Mesh	Coll.	Catalog #	Valve	Family	Genus (Subgenus)	Species	Authority	Reg.	Locality	Lat	Long	Scan Res (µm)
1221	USNM	631403	L	Tellinidae	*Scissula*	*candeana*	(d'Orbigny 1853)	FK	Grassy Key, Florida Keys, USA	24.76	-80.95	29.77
1027	FMNHIZ	41761	L	Tellinidae	*Scissula*	*similis*	(Sowerby 1806)	FK	† Harrington Sound, Bermuda	32.33	-64.72	31.17
1198	USNM	249980	L	Tellinidae	*Scissula*	*similis*	(Sowerby 1806)	FK	Elliott Key, Florida Keys, USA	25.44	-80.20	31.09
218	FMNHIZ	190931	L	Tellinidae	*Serratina*	*aequistriata*	(Say 1824)	FK	Missouri Key, Florida Keys, USA	24.68	-81.24	27.63
1038	FMNHIZ	82266	L	Tellinidae	*Serratina*	*martinicensis*	(d'Orbigny in Sagra 1853)	FK	† Nassau, New Providence Island, Bahamas	25.04	-77.40	31.17
229	FMNHIZ	202818	L	Tellinidae	*Strigilla*	*carnaria*	(Linnaeus 1758)	FK	Bahia Honda Key, Florida Keys, USA	24.67	-81.26	27.63
1100	USNM	185470a	L	Tellinidae	*Strigilla*	*gabbi*	Olsson & McGinty 1958	FK	† Puerto Limon, Costa Rica	10.01	-83.03	30.50
224	FMNHIZ	189417	L	Tellinidae	*Strigilla (Pisostrigilla)*	*mirabilis*	(Philippi 1841)	FK	Bahia Honda Key, Florida Keys, USA	24.67	-81.26	27.63
222	FMNHIZ	191018	L	Tellinidae	*Strigilla (Pisostrigilla)*	*pisiformis*	(Linnaeus 1758)	FK	Missouri Key, Florida Keys, USA	24.68	-81.24	27.63
1298	USNM	669771	L	Tellinidae	*Tampaella*	*leei*	Huber 2015	FK	Missouri Key, Florida Keys, USA	24.68	-81.24	30.80
216	FMNHIZ	311630	L	Tellinidae	*Tampaella*	*mera*	(Say 1834)	FK	Florida Keys, USA	24.76	-80.98	27.63
1211	USNM	53640	L	Tellinidae	*Tampaella*	*paramera*	(Boss 1964)	FK	Key West, Florida Keys, USA	24.55	-81.78	31.09
177	FMNHIZ	182765	L	Tellinidae	*Tampaella*	*tampaensis*	(Conrad 1866)	FK	Key West, Florida Keys, USA	24.55	-81.78	38.98
906	FMNHIZ	177480	L	Tellinidae	*Tellidora*	*cristata*	(Recluz 1842)	FK	† Bradenton Beach, West Florida, USA	27.47	-82.70	33.33
266	FMNHIZ	208420	L	Tellinidae	*Tellina*	*radiata*	Linnaeus 1758	FK	† Sanibel Island, West Florida, USA	26.43	-82.12	35.70
274	FMNHIZ	319801	L	Tellinidae	*Tellinella*	*listeri*	(Roeding 1798)	FK	† San Blas, Panama	9.58	-78.76	34.04

Mesh	Coll.	Catalog #	Valve	Family	Genus (Subgenus)	Species	Authority	Reg.	Locality	Lat	Long	Scan Res (µm)
1601	USNM	124104	L	Tellinidae	*Tellinella*	*mexicana*	(Petit de la Saussaye 1841)	FK	† St Helena, South Carolina, USA	32.40	-80.56	59.40
231	FMNHIZ	227474	L	Thraciidae	*Asthenothaerus*	*hemphillii*	Dall 1886	FK	Missouri Key, Florida Keys, USA	24.68	-81.24	27.63
1230	USNM	87523	R	Thraciidae	*Bushia*	*elegans*	(Dall 1886)	FK	Gulf of Mexico, Florida Strait, USA	24.22	-81.76	29.77
1615	USNM	784693	L	Thraciidae	*Cyathodonta*	*rugosa*	(Lamarck 1818)	FK	† Paraguaná Peninsula, Venezuela	11.60	-70.01	59.40
1041	USNM	485625	L	Thraciidae	*Thracia (Homoeodesma)*	*stimpsoni*	Dall 1886	FK	† Charleston, South Carolina, USA	32.77	-79.94	51.90
1371	USNM	53692	L	Thraciidae	*Thracia (Ixartia)*	*morrisoni*	Petit 1964	FK	Key West, Florida Keys, USA	24.55	-81.78	30.66
341	FMNHIZ	203552	R	Thyasiridae	*Thyasira*	*trisinuata*	(d'Orbigny 1853)	FK	† Lantana, East Florida, USA	26.58	-80.05	14.68
192	FMNHIZ	188801	L	Trapezidae	*Coralliophaga*	*coralliophaga*	(Gmelin 1791)	FK	Garden Key, Dry Tortugas, Florida Keys, USA	24.63	-82.87	38.98
807	FMNHIZ	187146	L	Ungulinidae	*Diplodonta*	*guaraniana*	(d'Orbigny 1846)	FK	† Nassau, New Providence Island, Bahamas	25.04	-77.40	31.92
288	FMNHIZ	185027	R	Ungulinidae	*Diplodonta*	*notata*	(Dall & Simpson 1901)	FK	† Tampa Bay, West Florida, USA	27.76	-82.54	28.20
212	FMNHIZ	176494	L	Ungulinidae	*Diplodonta*	*punctata*	(Say 1822)	FK	Missouri Key, Florida Keys, USA	24.68	-81.24	27.63
1209	USNM	102905	L	Ungulinidae	*Felaniella*	*candeana*	(d'Orbigny 1853)	FK	† Trinidad	10.37	-61.25	31.09
496	FMNHIZ	311582	L	Ungulinidae	*Foveamysia*	*soror*	(C. B. Adams 1852)	FK	Bethel Bank, Florida Keys, USA	24.73	-81.13	21.82
232	FMNHIZ	26370	L	Ungulinidae	*Phlyctiderma*	*semiasperum*	(Philippi 1836)	FK	Missouri Key, Florida Keys, USA	24.68	-81.24	27.63
874	FMNHIZ	82268	L	Ungulinidae	*Timothynus*	*rehderi*	(Altena 1968)	FK	† Nassau, New Providence	25.04	-77.40	29.30

Mesh	Coll.	Catalog #	Valve	Family	Genus (Subgenus)	Species	Authority	Reg.	Locality	Lat	Long	Scan Res (µm)
									Island, Bahamas			
184	FMNHIZ	176386	L	Veneridae	*Anomalocardia*	*cuneimeris*	(Conrad 1846)	FK	Key West, Florida Keys, USA	24.55	-81.78	38.98
1169	USNM	653508	L	Veneridae	*Anomalocardia*	*puella*	(Pfeiffer in Philippi 1846)	FK	† Sanibel Island, West Florida, USA	26.43	-82.12	34.22
469	FMNHIZ	306339	L	Veneridae	*Callista (Costacallista)*	*eucymata*	(Dall 1890)	FK	† Alcobaça, State of Bahia, Brazil	-17.55	39.19	78.93
180	FMNHIZ	176349	L	Veneridae	*Chione*	*elevata*	(Say 1822)	FK	Bonefish Key, Florida Keys, USA	24.73	-81.03	38.98
226	FMNHIZ	190789	L	Veneridae	*Chione*	*mazyckii*	Dall 1902	FK	Missouri Key, Florida Keys, USA	24.68	-81.24	27.63
870	FMNHIZ	176307	L	Veneridae	*Chioneryx*	*grus*	(Holmes 1858)	FK	† Bradenton Beach, West Florida, USA	27.47	-82.70	29.30
511	FMNHIZ	280845	L	Veneridae	*Chioneryx*	*pygmaea*	(Lamarck 1818)	FK	† NE of Nassau, New Providence Island, Bahamas	25.07	-77.28	30.78
401	FMNHIZ	283524	L	Veneridae	*Chionopsis (Puberella) [vii]*	*crenata*	(Gmelin 1791)	FK	† Santos, Sao Paulo, Brazil	-23.95	-46.32	56.47
205	FMNHIZ	121622	L	Veneridae	*Chionopsis (Puberella) [vii]*	*intapurpurea*	(Conrad 1849)	FK	Key West, Florida Keys, USA	24.55	-81.78	59.43
176	FMNHIZ	188788	L	Veneridae	*Choristodon*	*robustus*	(Sowerby 1834)	FK	Bonefish Key, Florida Keys, USA	24.73	-81.03	38.98
282	FMNHIZ	202928	L	Veneridae	*Cooperella*	*atlantica*	Rehder 1943	FK	Lake Worth, East Florida, USA	26.62	-80.04	28.20
220	FMNHIZ	306070	L	Veneridae	*Cyclinella*	*tenuis*	(Recluz 1852)	FK	Florida Keys, USA	24.76	-80.98	27.63
383	FMNHIZ	77993	L	Veneridae	*Dosinia*	*concentrica*	(Born 1778)	FK	† Sanibel Island, West Florida, USA	26.43	-82.12	58.79
200	FMNHIZ	185255	L	Veneridae	*Dosinia*	*discus*	(Reeve 1850)	FK	Key West, Florida Keys, USA	24.55	-81.78	59.43

Mesh	Coll.	Catalog #	Valve	Family	Genus (Subgenus)	Species	Authority	Reg.	Locality	Lat	Long	Scan Res (µm)
490	FMNHIZ	16679	R	Veneridae	*Gemma*	*gemma*	(Totten 1834)	FK	† Point Pinos, Pacific Grove, California, USA	36.64	-121.93	21.82
405	FMNHIZ	306324	L	Veneridae	*Globivenus*	*rigida*	(Dillwyn 1817)	FK	† Sao Paulo, Brazil	-21.81	-49.54	55.39
367	FMNHIZ	296332	L	Veneridae	*Globivenus*	*rugatina*	(Heilprin 1887)	FK	† Portobelo, Panama	9.55	-79.66	58.83
1108	USNM	656168	L	Veneridae	*Globivenus*	*strigillina*	(Dall 1902)	FK	Florida Straits, Florida, USA	24.22	-81.76	36.55
1215	USNM	821432	L	Veneridae	*Gouldia*	*cerina*	(C.B. Adams 1845)	FK	† Cape Canaveral, Florida, USA	28.39	-80.62	31.09
395	FMNHIZ	78386	L	Veneridae	*Hysteroconcha*	*dione*	(Linnaeus 1758)	FK	† Cartagena, Colombia	10.40	-75.53	45.57
276	FMNHIZ	78390	R	Veneridae	*Lamelliconcha*	*circinata*	(Born 1778)	FK	† Cartagena, Colombia	10.40	-75.53	34.04
188	FMNHIZ	176350	L	Veneridae	*Lirophora*	*latilirata*	(Conrad 1841)	FK	Key West, Florida Keys, USA	24.55	-81.78	38.98
594	FMNHIZ	155603	L	Veneridae	*Lirophora*	*obliterata*	Dall 1902	FK	† Port Isabel, Texas, USA	26.08	-97.21	39.92
543	FMNHIZ	176342	L	Veneridae	*Lirophora*	*paphia*	(Linnaeus 1767)	FK	† Havana, Cuba	23.15	-82.37	57.49
197	FMNHIZ	166375	L	Veneridae	*Macrocallista*	*nimbosa*	(Lightfoot 1786)	FK	Dry Tortugas, Florida Keys, USA	24.63	-82.87	59.43
203	FMNHIZ	176333	L	Veneridae	*Megapitaria*	*maculata*	(Linnaeus 1758)	FK	Key West, Florida Keys, USA	24.55	-81.78	59.43
209	FMNHIZ	223476	L	Veneridae	*Mercenaria*	*campechiensis*	(Gmelin 1791)	FK	† St Petersburg, West Florida, USA	27.78	-82.66	104.24
334	FMNHIZ	185769	R	Veneridae	*Parastarte*	*triquetra*	(Conrad 1846)	FK	† Tampa Bay, West Florida, USA	27.76	-82.54	14.68
199	FMNHIZ	301426	L	Veneridae	*Periglypta*	*listeri*	(Gray 1838)	FK	"The Horseshoe" reef off Marathon, Florida Keys, USA	24.65	-81.26	59.43
191	FMNHIZ	189224	L	Veneridae	*Petricola*	*lapicida*	(Gmelin 1791)	FK	Bonefish Key, Florida Keys, USA	24.73	-81.03	38.98

Mesh	Coll.	Catalog #	Valve	Family	Genus (Subgenus)	Species	Authority	Reg.	Locality	Lat	Long	Scan Res (µm)
239	FMNHIZ	188665	L	Veneridae	*Petricolaria*	*pholadiformis*	(Lamarck 1818)	FK	Key West, Florida Keys, USA	24.55	-81.78	39.54
540	FMNHIZ	158478	L	Veneridae	*Pitar*	*albidus*	(Gmelin 1791)	FK	† St Thomas, Virgin Islands	18.35	-64.90	57.49
251	FMNHIZ	26422	L	Veneridae	*Pitar*	*fulminatus*	(Menke 1828)	FK	† Lake Worth, East Florida, USA	26.62	-80.04	57.32
221	FMNHIZ	311594	L	Veneridae	*Pitar*	*simpsoni*	(Dall 1895)	FK	Florida Keys, USA	24.76	-80.98	27.63
254	FMNHIZ	41700	L	Veneridae	*Pitarenus*	*cordatus*	(Schwengel 1951)	FK	† Aransas Pass, Texas, USA	27.87	-97.12	57.32
1205	USNM	92008	R	Veneridae	*Pitarenus*	*zonatus*	(Dall 1902)	FK	† Cape Lookout, North Carolina, USA	34.61	-76.54	31.09
695	FMNHIZ	306330	L	Veneridae	*Tivela*	*mactroides*	(Born 1778)	FK	† Itajaí, Santa Catarina, Brazil	-26.92	-48.64	59.36
294	FMNHIZ	185690	R	Veneridae	*Tivela*	*trigonella*	(Lamarck 1818)	FK	† Virgin Islands	18.03	-64.75	28.20
292	FMNHIZ	208369	L	Veneridae	*Transennella*	*conradina*	Dall 1884	FK	† Sanibel Island, West Florida, USA	26.43	-82.12	28.20
351	FMNHIZ	311604	R	Veneridae	*Transennella*	*cubaniana*	(d'Orbigny 1853)	FK	Key Colony Beach, Florida Keys, USA	24.72	-81.02	15.79
2116	UFIZ	143402	R	Veneridae	*Transennella*	*culebrana*	(Dall & Simpson 1901)	FK	† Altona Bay, Virgin Islands	17.72	-64.82	29.21
508	FMNHIZ	283508	L	Veneridae	*Transennella*	*stimpsoni*	Dall 1902	FK	† St Augustine, East Florida, USA	29.90	-81.32	30.78
1242	USNM	444367	R	Verticordiidae	*Haliris*	*fischeriana*	(Dall 1881)	FK	Key West, Florida Keys, USA	24.55	-81.78	29.77
358	FMNHIZ	26387	L	Verticordiidae	*Trigonulina*	*ornata*	d'Orbigny 1846	FK	† Destin, Northwest Florida, USA	30.39	-86.50	15.79
1310	USNM	95797	R	Yoldiidae	*Orthoyoldia*	*liorhina*	(Dall 1881)	FK	† Barbados	13.23	-59.53	20.71
592	FMNHIZ	2371	L	Anomiidae	*Anomia*	*simplex*	d'Orbigny 1853	GM	Buzzard's Bay, Massachusetts, USA	41.74	-70.62	39.92

Mesh	Coll.	Catalog #	Valve	Family	Genus (Subgenus)	Species	Authority	Reg.	Locality	Lat	Long	Scan Res (µm)
631	FMNHIZ	59436	L	Arcticidae	*Arctica*	*islandica*	(Linnaeus 1767)	GM	Penobscot Bay, Maine, USA	44.46	-68.72	77.94
1093	USNM	76510	L	Astartidae	*Astarte*	*borealis*	(Schumacher 1817)	GM	Massachusetts Bay, USA	42.32	-70.79	37.60
3579	FMNHIZ	13759	L	Astartidae	*Astarte*	*montagui*	(Dillwyn 1817)	GM	† Shoal Tickle, Anatalak Bay, Nain, Labrador	56.58	-61.58	34.77
1091	USNM	76525	L	Astartidae	*Astarte*	*subaequilatera*	Sowerby II, 1854	GM	Massachusetts Bay, USA	42.32	-70.79	37.60
1072	USNM	273453	L	Astartidae	*Astarte*	*undata*	Gould 1841	GM	Nova Scotia, Canada	45.05	-62.70	31.86
641	FMNHIZ	54723	L	Astartidae	*Isocrassina[viii]*	*castanea*	(Say 1822)	GM	Massachusetts Bay, USA	42.32	-70.79	40.21
565	FMNHIZ	163330	R	Cardiidae	*Ciliatocardium*	*ciliatum*	(Fabricius 1780)	GM	Gaspé Peninsula, Canada	48.67	-65.83	62.28
687	FMNHIZ	176781	R	Cardiidae	*Parvicardium*	*pinnulatum*	(Conrad 1831)	GM	Georges Bank, Massachusetts Bay, USA	40.91	-68.53	22.26
1101	USNM	794759	L	Carditidae	*Cyclocardia*	*borealis*	(Conrad 1831)	GM	Cape Cod Lighthouse, Massachusetts Bay, USA	41.93	-70.00	30.50
1703	USNM	781525	L	Carditidae	*Cyclocardia*	*novangliae*	(Morse 1869)	GM	Cape Cod Lighthouse, Massachusetts Bay, USA	41.93	-70.00	36.45
864	FMNHIZ	190650	L	Corbulidae	*Caryocorbula*	*contracta*	(Say 1822)	GM	Quisset Harbour, Massachusetts, USA	41.54	-70.66	29.30
869	FMNHIZ	190650	R	Corbulidae	*Caryocorbula*	*contracta*	(Say 1822)	GM	Quisset Harbour, Massachusetts, USA	41.54	-70.66	29.30
1273	USNM	49051	R	Cuspidariidae	*Cuspidaria*	*glacialis*	(Sars 1878)	GM	Cape Cod, Massachusetts, USA	41.68	-70.30	29.77
1400	USNM	48984	L	Cuspidariidae	*Cuspidaria*	*obesa*	(Lovén 1846)	GM	Cape Cod, Massachusetts, USA	41.68	-70.30	33.39
1129	USNM	153691	L	Hiatellidae	*Cyrtodaria*	*siliqua*	(Spengler 1793)	GM	Massachusetts Bay, USA	42.32	-70.79	53.95

Mesh	Coll.	Catalog #	Valve	Family	Genus (Subgenus)	Species	Authority	Reg.	Locality	Lat	Long	Scan Res (µm)
678	FMNHIZ	2855	R	Hiatellidae	*Hiatella*	*arctica*	(Linnaeus 1767)	GM	Nova Scotia, Canada	45.05	-62.70	22.26
1681	USNM	49945	L	Hiatellidae	*Panomya*	*norvegica*	(Spengler 1793)	GM	Cape Cod, Massachusetts, USA	41.68	-70.30	55.83
1216	USNM	35024	L	Limopsidae	*Limopsis*	*sulcata*	Verrill & Bush 1898	GM	Martha's Vineyard, Massachusetts, USA	41.38	-70.64	31.09
1282	USNM	41359	L	Lucinidae	*Divalinga*	*strigilla*	(Stimpson 1851)	GM	Nahant Bay, Massachusetts, USA	42.45	-70.91	33.44
685	FMNHIZ	185735	L	Lyonsiidae	*Lyonsia*	*hyalina*	(Conrad 1831)	GM	Old Orchard Beach, Maine, USA	43.52	-70.37	22.26
1694	USNM	74536	L	Mactridae	*Mactromeris*	*polynyma*	(Stimpson 1860)	GM	Cape Cod, Massachusetts, USA	41.68	-70.30	82.82
691	FMNHIZ	59617	L	Mactridae	*Mulinia*	*lateralis*	(Say 1822)	GM	Massachusetts Bay, USA	42.32	-70.79	22.26
1107	USNM	37020	L	Mactridae	*Spisula (Hemimactra)*	*solidissima*	(Dillwyn 1817)	GM	Massachusetts Bay, USA	42.32	-70.79	36.55
1064	USNM	95578	R	Mesodesmatidae	*Mesodesma*	*arctatum*	(Conrad 1831)	GM	Rockport, Massachusetts, USA	42.67	-70.62	33.47
547	FMNHIZ	169274	L	Myidae	*Mya*	*arenaria*	(Linnaeus 1758)	GM	Cape Cod, Massachusetts, USA	41.68	-70.30	62.28
1084	USNM	27368	L	Myidae	*Mya*	*truncata*	Linnaeus 1758	GM	Nahant Bay, Massachusetts, USA	42.45	-70.91	37.60
1303	USNM	73987	R	Mytilidae	*Crenella*	*decussata*	(Montagu 1808)	GM	Cape Cod, Massachusetts, USA	41.68	-70.30	20.71
572	FMNHIZ	171894	L	Mytilidae	*Geukensia*	*demissa*	(Dillwyn 1817)	GM	† Cape May, New Jersey, USA	38.93	-74.90	58.40
673	FMNHIZ	155519	L	Mytilidae	*Ischadium*	*recurvum*	(Rafinesque 1820)	GM	† Rhode Island, USA	41.59	-71.40	29.10
570	FMNHIZ	126621	R	Mytilidae	*Modiolus*	*modiolus*	(Linnaeus 1758)	GM	Nova Scotia, Canada	45.05	-62.70	58.40
1074	USNM	73720	L	Mytilidae	*Musculus*	*discors*	(Linnaeus 1767)	GM	Cape Cod, Massachusetts, USA	41.68	-70.30	31.86

Mesh	Coll.	Catalog #	Valve	Family	Genus (Subgenus)	Species	Authority	Reg.	Locality	Lat	Long	Scan Res (µm)
1609	USNM	73779	R	Mytilidae	*Musculus*	*niger*	(Gray 1824)	GM	† Martha's Vineyard, Massachusetts, USA	41.40	-70.66	59.40
575	FMNHIZ	140710	R	Mytilidae	*Mytilus*	*edulis*	Linnaeus 1758	GM	Cape Cod, Massachusetts, USA	41.68	-70.30	58.40
1264	USNM	73937	L	Mytilidae	*Solamen*	*glandula*	(Totten 1834)	GM	Cape Cod, Massachusetts, USA	41.68	-70.30	29.77
1299	USNM	1446647	R	Nuculanidae	*Nuculana*	*tenuisulcata*	(Couthouy 1838)	GM	Georges Bank, Massachusetts Bay, USA	40.91	-68.53	20.71
682	FMNHIZ	148375	L	Nuculidae	*Ennucula*	*delphinodonta*	(Mighels & Adams 1842)	GM	Maine, USA	44.17	-68.66	22.26
689	FMNHIZ	12729	L	Nuculidae	*Ennucula*	*tenuis*	(Montagu 1808)	GM	Labrador, Canada	53.26	-58.74	22.26
680	FMNHIZ	185387	L	Nuculidae	*Nucula*	*proxima*	Say 1822	GM	Maine, USA	44.17	-68.66	22.26
1430	USNM	49759	L	Pandoridae	*Clidiophora*	*inornata*	Verrill & Bush 1898	GM	Cape Cod, Massachusetts, USA	41.68	-70.30	30.91
1292	USNM	444667	R	Pandoridae	*Pandora (Pandorella)*	*glacialis*	Leach 1819	GM	Labrador, Canada	53.26	-58.74	30.80
1261	USNM	40232	L	Pandoridae	*Pandora (Pandorella)*	*inflata*	Boss & Merrill 1965	GM	Nantucket, Massachusetts, USA	41.27	-70.05	29.77
551	FMNHIZ	183565	R	Pectinidae	*Argopecten*	*irradians irradians*	(Lamarck 1819)	GM	Cape Cod, Massachusetts, USA	41.68	-70.30	62.28
577	FMNHIZ	183565	L	Pectinidae	*Argopecten*	*irradians irradians*	(Lamarck 1819)	GM	Cape Cod, Massachusetts, USA	41.68	-70.30	58.40
566	FMNHIZ	163336	L	Pectinidae	*Chlamys*	*islandica*	(Müller 1776)	GM	Gaspé Peninsula, Canada	48.67	-65.83	62.28
1344	USNM	764667	L	Pectinidae	*Delectopecten*	*vitreus*	(Gmelin 1791)	GM	Martha's Vineyard, Massachusetts, USA	41.38	-70.64	35.68
1394	USNM	764667	R	Pectinidae	*Delectopecten*	*vitreus*	(Gmelin 1791)	GM	Martha's Vineyard, Massachusetts, USA	41.38	-70.64	33.39
3580	FMNHIZ	6140	L	Pectinidae	*Palliolum*	*striatum*	(Müller 1776)	GM	†British Isles	53.68	-3.81	34.77

Mesh	Coll.	Catalog #	Valve	Family	Genus (Subgenus)	Species	Authority	Reg.	Locality	Lat	Long	Scan Res (µm)
633	FMNHIZ	126630	L	Pectinidae	*Placopecten*	*magellanicus*	(Gmelin 1791)	GM	Cape Cod, Massachusetts, USA	41.68	-70.30	77.94
822	FMNHIZ	188773	L	Periplomatidae	*Cochlodesma*	*leanum*	(Conrad 1831)	GM	Cape Cod, Massachusetts, USA	41.68	-70.30	31.92
1700	USNM	202870	L	Periplomatidae	*Septentrioploma*	*aleuticum*	(Krause 1885)	GM	† Halifax, Nova Scotia	44.63	-63.55	36.45
1705	USNM	44769	R	Periplomatidae	*Septentrioploma*	*fragile*	(Totten 1835)	GM	Golls Island, Maine, USA	44.20	-68.42	36.45
613	FMNHIZ	150752	L	Pharidae	*Ensis*	*leei*	Huber 2015	GM	Saugus River, Massachusetts, USA	42.52	-71.07	98.70
1080	USNM	462343	L	Pharidae	*Siliqua*	*costata*	(Say 1822)	GM	Massachusetts Bay, USA	42.32	-70.79	31.86
1090	USNM	462635	L	Pholadidae	*Zirfaea*	*crispata*	Linnaeus 1758	GM	Duxbury, Massachusetts, USA	42.02	-70.68	37.60
1378	USNM	50684	R	Propeamussiidae	*Cyclopecten*	*hoskynsi*	(Forbes 1844)	GM	Nova Scotia, Canada	45.05	-62.70	30.66
1379	USNM	62264	R	Propeamussiidae	*Cyclopecten*	*hoskynsi*	(Forbes 1844)	GM	Nova Scotia, Canada	45.05	-62.70	30.66
1255	USNM	52170	L	Semelidae	*Abra*	*longicallus*	(Scacchi 1835)	GM	† Martha's Vineyard, Massachusetts, USA	41.40	-70.66	29.77
1468	USNM	27071	L	Solemyidae	*Solemya (Petrasma)*	*borealis*	Totten 1834	GM	Massachusetts Bay, USA	42.32	-70.79	34.63
1698	USNM	131490	L	Solemyidae	*Solemya (Petrasma)*	*velum*	Say 1822	GM	Massachusetts Bay, USA	42.32	-70.79	36.45
684	FMNHIZ	177945	L	Tellinidae	*Ameritella*	*agilis*	(Stimpson 1857)	GM	Massachusetts Bay, USA	42.32	-70.79	22.26
643	FMNHIZ	59636	L	Tellinidae	*Limecola*	*balthica*	(Linnaeus 1758)	GM	Massachusetts Bay, USA	42.32	-70.79	40.21
892	FMNHIZ	71115	L	Tellinidae	*Macoma*	*calcarea*	(Gmelin 1791)	GM	Massachusetts Bay, USA	42.32	-70.79	31.06
898	FMNHIZ	158480	L	Tellinidae	*Macoma*	*petalum*	(Valenciennes 1827)	GM	Portland, Maine, USA	43.65	-70.26	31.06
1082	USNM	73623	R	Thraciidae	*Thracia (Crassithracia)*	*myopsis*	(Möller 1842)	GM	Cape Cod, Massachusetts, USA	41.68	-70.30	37.60
1259	USNM	444797	L	Thraciidae	*Thracia (Crassithracia)*	*septentrionalis*	Jeffreys 1872	GM	Frenchman’s Bay, Canada	43.82	-79.09	29.77

Mesh	Coll.	Catalog #	Valve	Family	Genus (Subgenus)	Species	Authority	Reg.	Locality	Lat	Long	Scan Res (µm)
1139	USNM	460763	L	Thraciidae	*Thracia (Homoeodesma)*	*conradi*	Couthouy 1839	GM	Duxbury, Massachusetts, USA	42.02	-70.68	56.59
3366	BMNH	1911102646017-23	L	Thyasiridae	*Axinopsida*	*orbiculata*	(Sars 1878)	GM	† Finnmark, Norway	71.06	26.45	30.15
1306	USNM	34871	R	Thyasiridae	*Mendicula*	*ferruginosa*	(Forbes 1844)	GM	Georges Bank, Massachusetts Bay, USA	40.91	-68.53	20.71
1307	USNM	74303	R	Thyasiridae	*Parathyasira*	*equalis*	(Verrill & Bush 1898)	GM	Gulf of Maine, USA	43.24	-68.36	20.71
1251	USNM	74263	R	Thyasiridae	*Thyasira*	*gouldii*	(Philippi 1845)	GM	Cape Cod, Massachusetts, USA	41.68	-70.30	29.77
683	FMNHIZ	26280	R	Veneridae	*Gemma*	*gemma*	(Totten 1834)	GM	Cape Cod, Massachusetts, USA	41.68	-70.30	22.26
654	FMNHIZ	77107	L	Veneridae	*Liocyma*	*fluctuosa*	(Gould 1841)	GM	† Kiska Harbor, Aleutians	51.97	177.60	40.21
635	FMNHIZ	3575	L	Veneridae	*Mercenaria*	*mercenaria*	(Linnaeus 1758)	GM	† Newport, Rhode Island, USA	41.48	-71.32	77.94
529	FMNHIZ	188668	L	Veneridae	*Petricolaria*	*pholadiformis*	(Lamarck 1818)	GM	Cape Cod, Massachusetts, USA	41.68	-70.30	57.49
553	FMNHIZ	3579	L	Veneridae	*Pitar*	*morrhuanus*	Dall 1902	GM	† Newport, Rhode Island, USA	41.48	-71.32	62.28
1436	USNM	24613	R	Veneridae	*Turtonia*	*minuta*	(Fabricius 1780)	GM	Massachusetts Bay, USA	42.32	-70.79	30.91
1054	USNM	51446	L	Yoldiidae	*Megayoldia*	*thraciaeformis*	(Storer 1838)	GM	Massachusetts Bay, USA	42.32	-70.79	33.47
1278	USNM	95801	L	Yoldiidae	*Yoldia*	*limatula*	(Say 1831)	GM	† Eastport, Maine, USA	44.90	-66.98	33.44
3243	BMNH	1911102641159-60	R	Yoldiidae	*Yoldia*	*myalis*	(Couthouy 1838)	GM	Nova Scotia, Canada	45.05	-62.70	35.03
1288	USNM	73241	L	Yoldiidae	*Yoldia*	*sapotilla*	(Gould 1841)	GM	Casco Bay, Maine, USA	43.70	-69.99	33.44
3338	BMNH	2E+07	L	Yoldiidae	*Yoldiella*	*frigida*	(Torell 1859)	GM	† Bergen Fjord, Norway	60.40	5.29	24.63
1311	USNM	74324	L	Yoldiidae	*Yoldiella*	*lucida*	(Lovén 1846)	GM	Casco Bay, Maine, USA	43.70	-69.99	20.71

[i] Potiarca is retained by Huber (2010) and found to be distinct molecularly from Anadara by Audino et al. (2019)

[ii] Vokesula is considered distinct from Varicorbula by Hallan et al. (2013)
[iii] Dacrydium (Quendreda) is considered distinct from Dacrydium s.s. by Coan & Valentich-Scott (2012: p.132)
[iv] An earlier name may be Ostrea capsa Fischer von Waldheim 1807
[v] Lindapecten is considered a synonym of Aequipecten by MolluscaBase but we retain it following Waller (2011) and H. H. Dijkstra (www.scallop.nl)
[vi] E. subcancellata is considered distinct from E. nitens by Marques & Simone (2011)
[vii] We retain Chionopsis (Puberella) as a subgenus following Roopnarine (2001) (see also Huber 2010: p.719)
[viii] Isocrassina is considered distinct from Astarte by Pouwer (2010)

References

Audino JA, Serb JM, Marian JEA. Ark clams and relatives (Bivalvia: Arcida) show convergent morphological evolution associated with lifestyle transitions in the marine benthos. Biol J Linn Soc. 2019;126: 866-884.

Coan EV, Valentich-Scott P. Bivalve seashells of tropical West America. Santa Barbara Museum of Natural History Monographs 6, 2 volumes. 2012.

Huber M. Compendium of bivalves. Hackenheim, Germany 2010; 901 p.

Hallan A, Colgan DJ, Anderson LC, Garcia A, Chivas AR. A single origin for the limnetic–euryhaline taxa in the Corbulidae (Bivalvia). Zool Scr. 2013; 42(3):278-287.

Marques RC, Simone LRL. A new species of *Ervilia* from north Brazil (Bivalvia, Semelidae). J Conch. 2011;40: 651-655.

Pouwer R. The identity of *Isocrassina*, *Laevastarte* and *Ashtarotha* (Mollusca, Bivalvia, Astartidae) and their representatives from beaches and estuaries in The Netherlands and Pliocene strata in Belgium. Cain Res. 2010;7: 27-67.

Roopnarine PD. A history of diversification, extinction, and invasion in tropical America as derived from species-level phylogenies of chionine genera (Family Veneridae). J Paleontol. 2001;75: 644-657.

Waller, TR. Neogene paleontology of the northern Dominican Republic. 24. Propeamussiidae and Pectinidae (Mollusca: Bivalvia: Pectinoidea) of the Cibao Valley. Bull Amer Paleontol 2011;381. 198 p.

Species recorded in the literature as being found in these regions that we were unable to include in analyses:

Family	Genus (Subgenus)	Species	Authority	Reg.
Poromyidae	*Cetomya*	*albida*	(Dall 1886)	FK
Chamidae	*Chama*	*inezae*	(Bayer 1943)	FK
Pholadidae	*Diplothyra*	*smithii*	Tryon 1862	FK
Nuculidae	*Ennucula*	*delphinodonta*	(Mighels & Adams 1842)	FK
Lucinidae	*Epicodakia*	*pectinata*	(C.B. Adams 1852	FK
Gastrochaenidae	*Gastrochaena*	*difficilis*	Deshayes 1855	FK
Mactridae	*Hemimactra*	*raveneli*	(Conrad 1832)	FK
Nuculanidae	*Ledella*	*sublaevis*	Verrill & Bush 1898	FK
Limidae	*Limaria*	*tuberculata*	(Olivi 1792)	FK
Tellinidae	*Macoma*	*cerina*	(C. B. Adams, 1845)	FK
Nuculidae	*Nucula*	*calcicola*	Moore 1977	FK
Astartidae	*Astarte*	*elliptica*	(Brown 1827)	GM
Astartidae	*Astarte*	*portlandica*	Mighels 1843	GM
Astartidae	*Astarte*	*quadrans*	Gould 1841	GM
Cuspidariidae	*Cuspidaria*	*pellucida*	(Stimpson 1853)	GM
Mytilidae	*Dacrydium*	*vitreum*	(Holboll in Möller 1842)	GM
Limidae	*Limatula*	*subauriculata*	(Montagu 1808)	GM
Lyonsiidae	*Lyonsia*	*arenosa*	(Möller 1842)	GM
Mytilidae	*Musculus*	*glacialis*	(Leche 1883)	GM

Periplomatidae	*Septentrioploma*	*papyratium*	(Say 1822)	GM
Myidae	*Sphenia*	*sincera*	Hanks & Packer 1985	GM
