# Supplementary material for: Spatial filters of function and phylogeny determine morphological disparity with latitude
Source: PLoS One. 2019 Aug 29;14(8):e0221490. doi: 10.1371/journal.pone.0221490 (PMC6715166; doi:10.1371/journal.pone.0221490)
Supplement: S2 Table — Tabulated results for Kolmogorov-Smirnov (MST distances) and Wilcoxon tests (disparities) between faunas and subgroups. (PDF) [file pone.0221490.s002.pdf]

Supplementary Data Table 2: Results of Kolmogorov-Smirnov tests on MST distances and Wilcoxon tests on disparity between regions for whole faunas and for subgroups (families and functional groups).

Group	KS statistic	p.value		Wilcoxon statistic	p.value	
Region	0.157	0.081		923	0.000	***
Astartidae	0.500	0.933		8065	0.000	***
Cardiidae	0.867	0.088		0	0.000	***
Mactridae	0.750	0.400		213	0.000	***
Mytilidae	0.619	0.026	***	5448	0.274	
Nuculidae	0.500	1.000		6565	0.000	***
Pectinidae	0.360	0.417		0	0.000	***
Tellinidae	0.326	0.839		0	0.000	***
Thraciidae	0.500	0.933		3251	0.000	***
Veneridae	0.833	0.004	***	9080	0.000	***
BY IM EP SUS	1.000	0.053		72	0.000	***
BY SW EP SUS	1.000	0.154		206	0.000	***
UN MO DIS CH	1.000	0.125		0	0.006	***
UN MO DIS SUS	1.000	0.143		6112	0.000	***
UN MO INA SBD	1.000	0.667		6282	0.000	***
UN MO INA SUS	1.000	0.105		0	0.000	***
UN MO SIS SBD	1.000	0.250		1023	0.981	
UN MO SIS SUS	1.000	0.031	***	5716	0.000	***
