# Supplementary figures and images for: Spatial filters of function and phylogeny determine morphological disparity with latitude
Source: PLoS One. 2019 Aug 29;14(8):e0221490. doi: 10.1371/journal.pone.0221490 (PMC6715166; doi:10.1371/journal.pone.0221490)

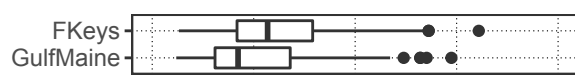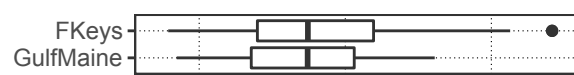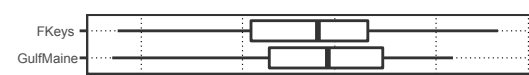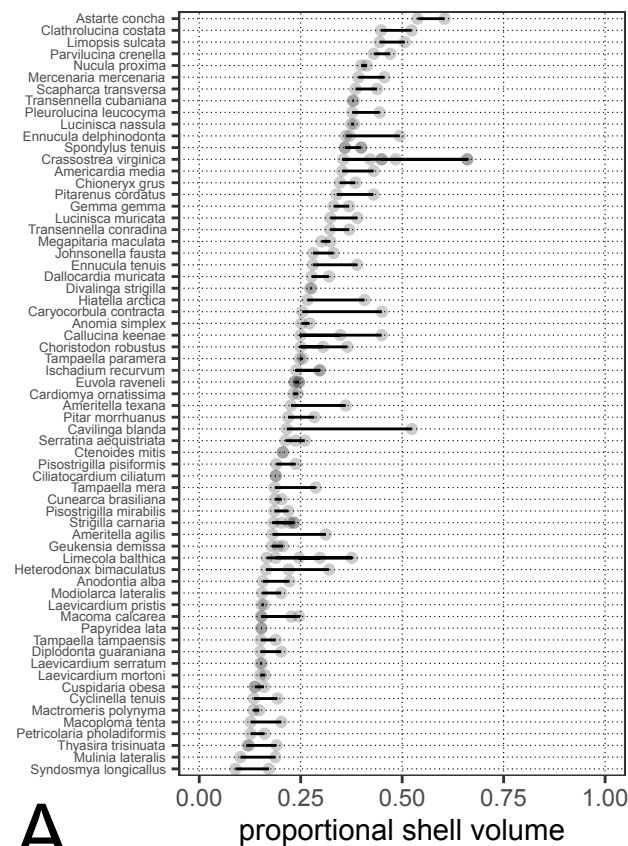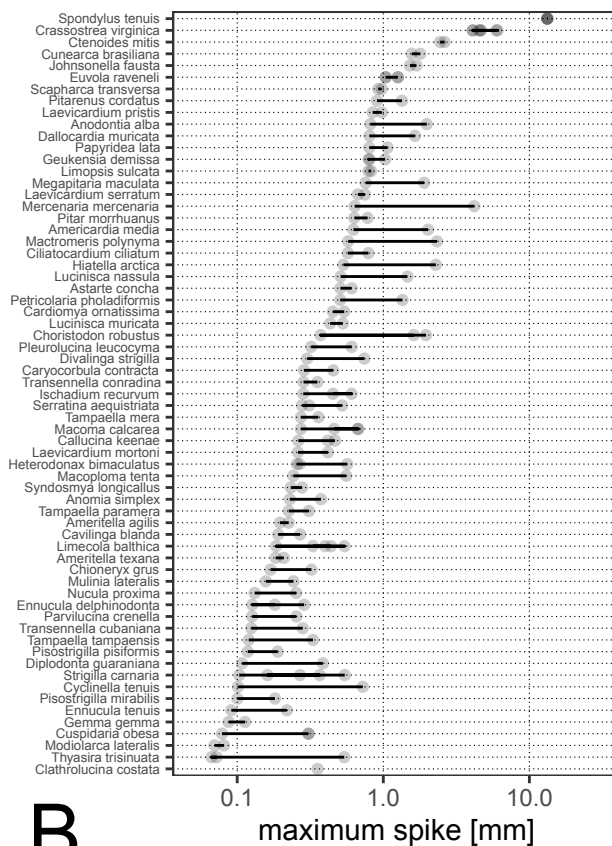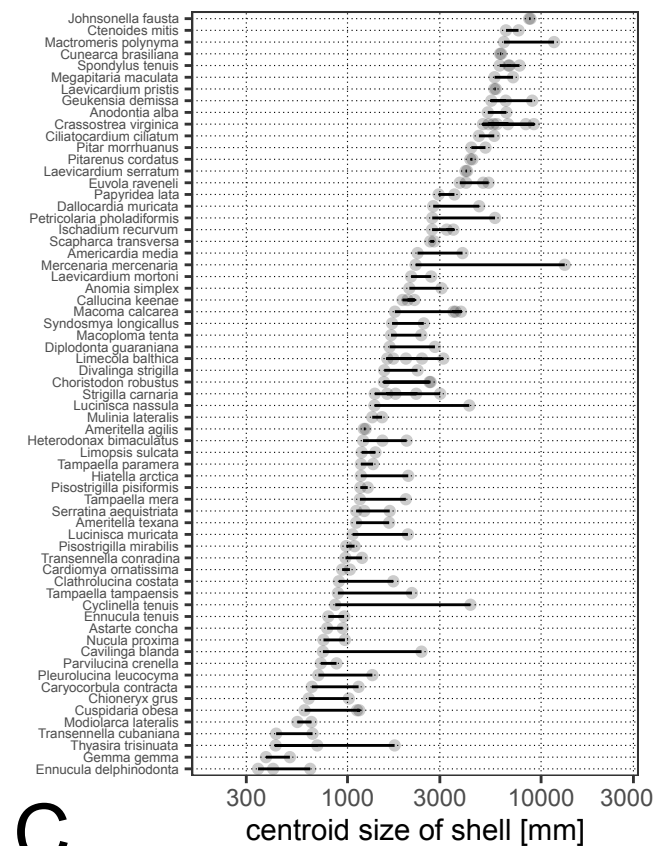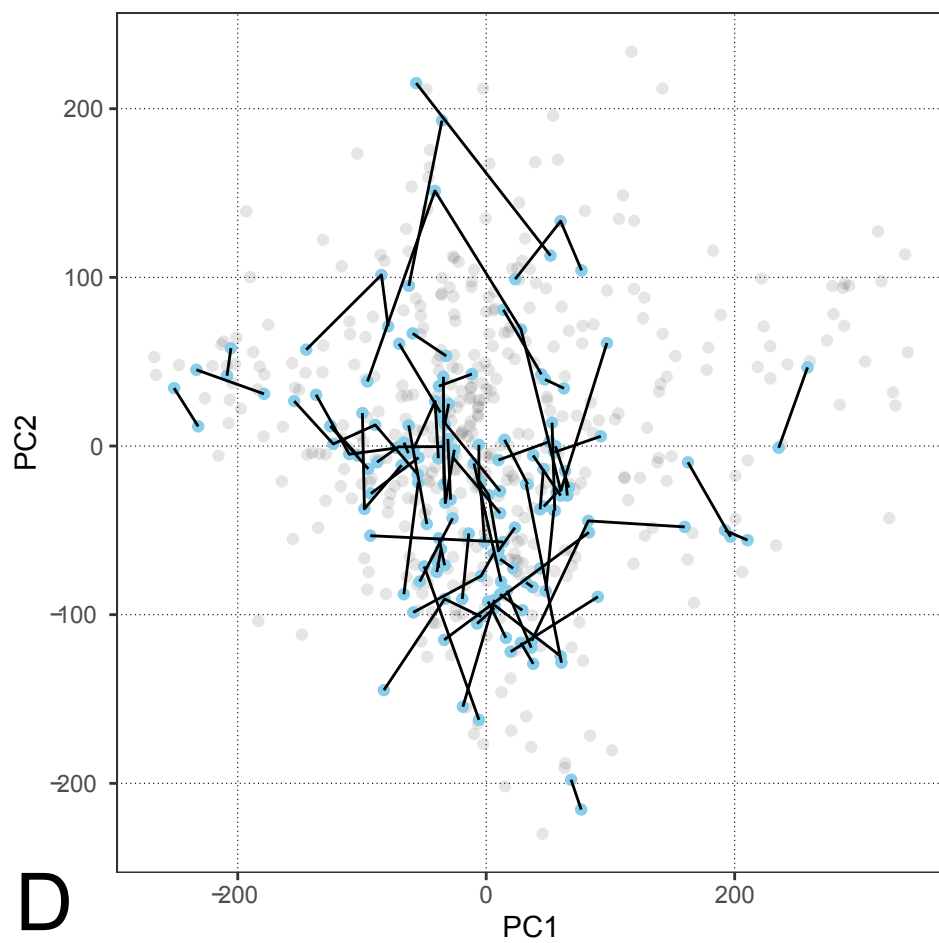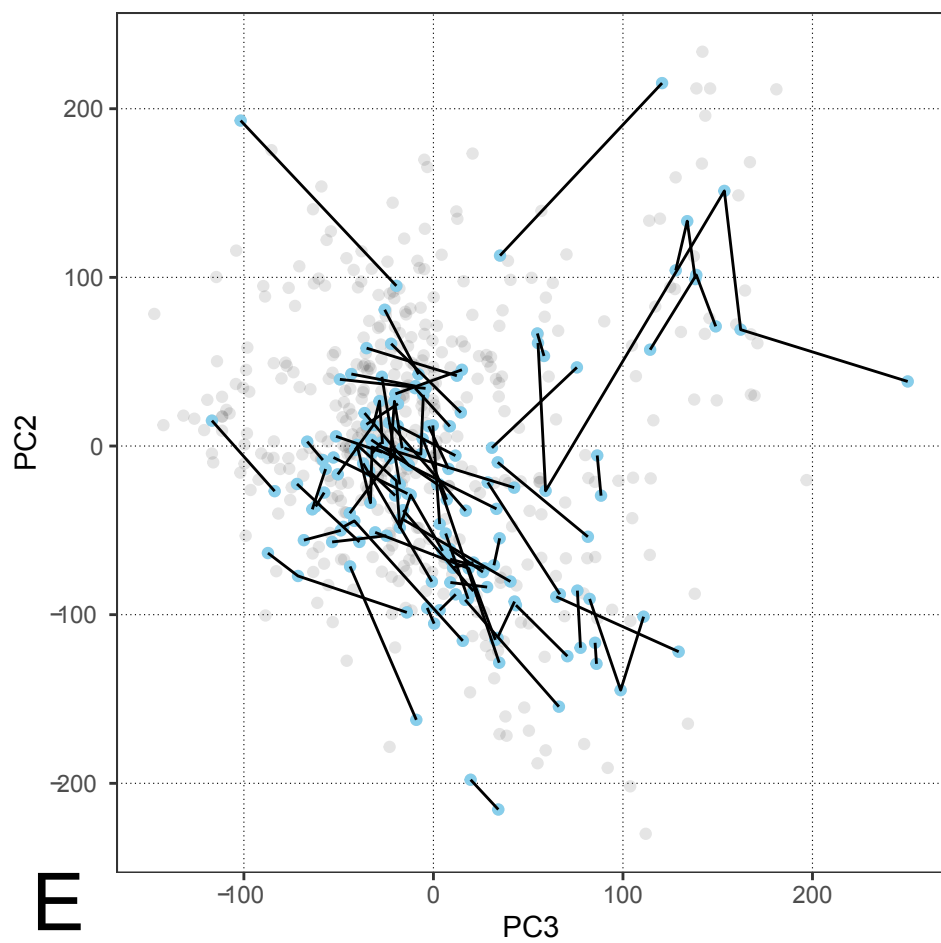

Supplement: S1 Fig — Comparison of 65 FK or GM species with more than 2 individuals scanned (for a wider, as-yet unpublished analysis of bivalve morphology–this dataset includes the FK or GM specimens of these species, plus additional specimens from localities outside of FK or GM which are not included in the main analysis of the paper) for the variables studied in this analysis. Panels A, B, C show species ranges for the three univariate traits. Panels D, E, show the bivariate plots of PCs 1+2, and 2+3 respectively, with the total FK-GM dataset plotted in grey behind the intraspecific variation dataset. Note that most species occupy narrow ranges of values for these traits compared to the overall range of the dataset. (PDF) [file pone.0221490.s003.pdf]

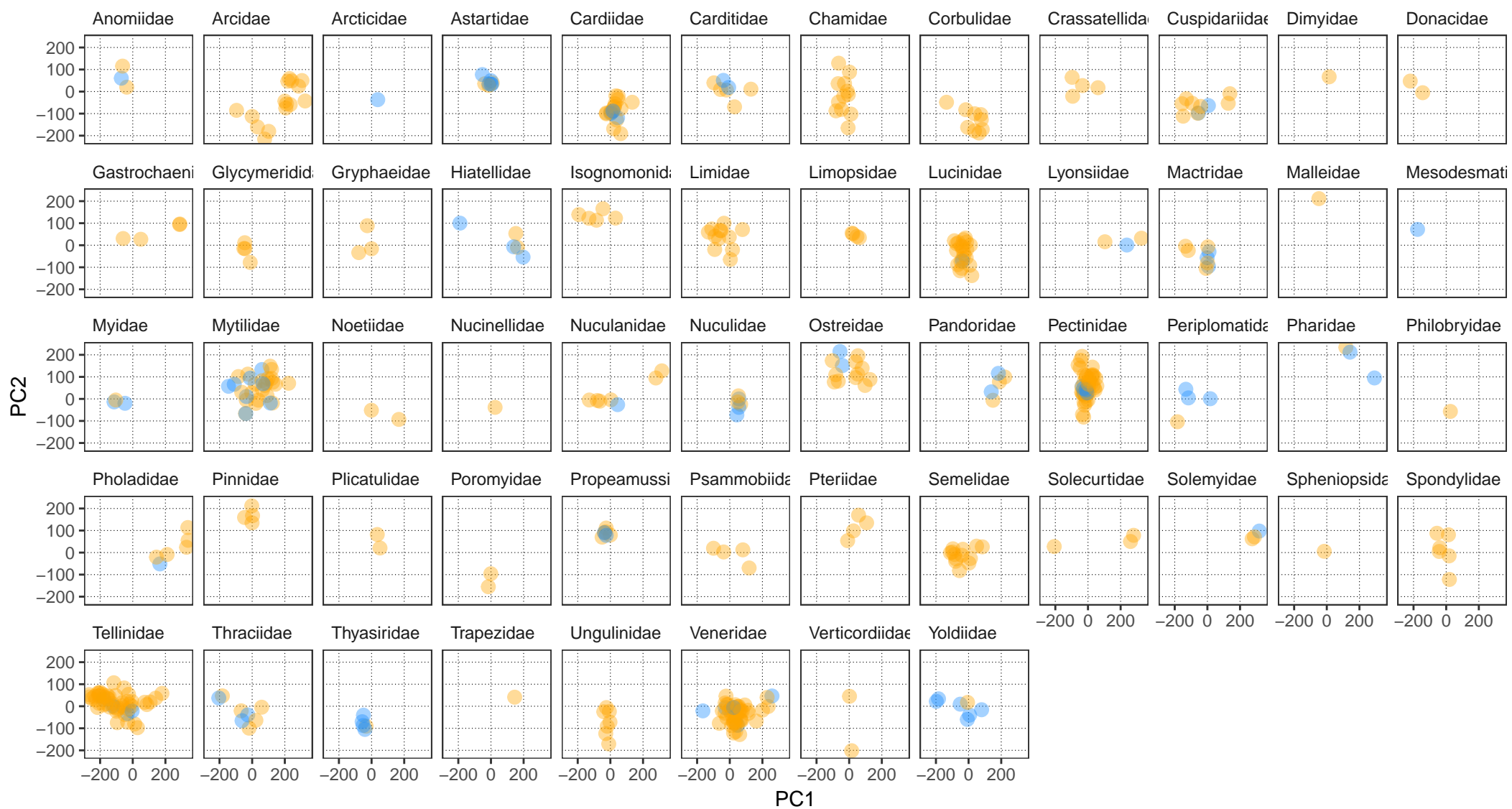

Supplement: S2 Fig — Multipanel figure displaying the same data as in Text Fig 3 but faceted by families in order to clarify within-subgroup patterns. (PDF) [file pone.0221490.s004.pdf]

PC2

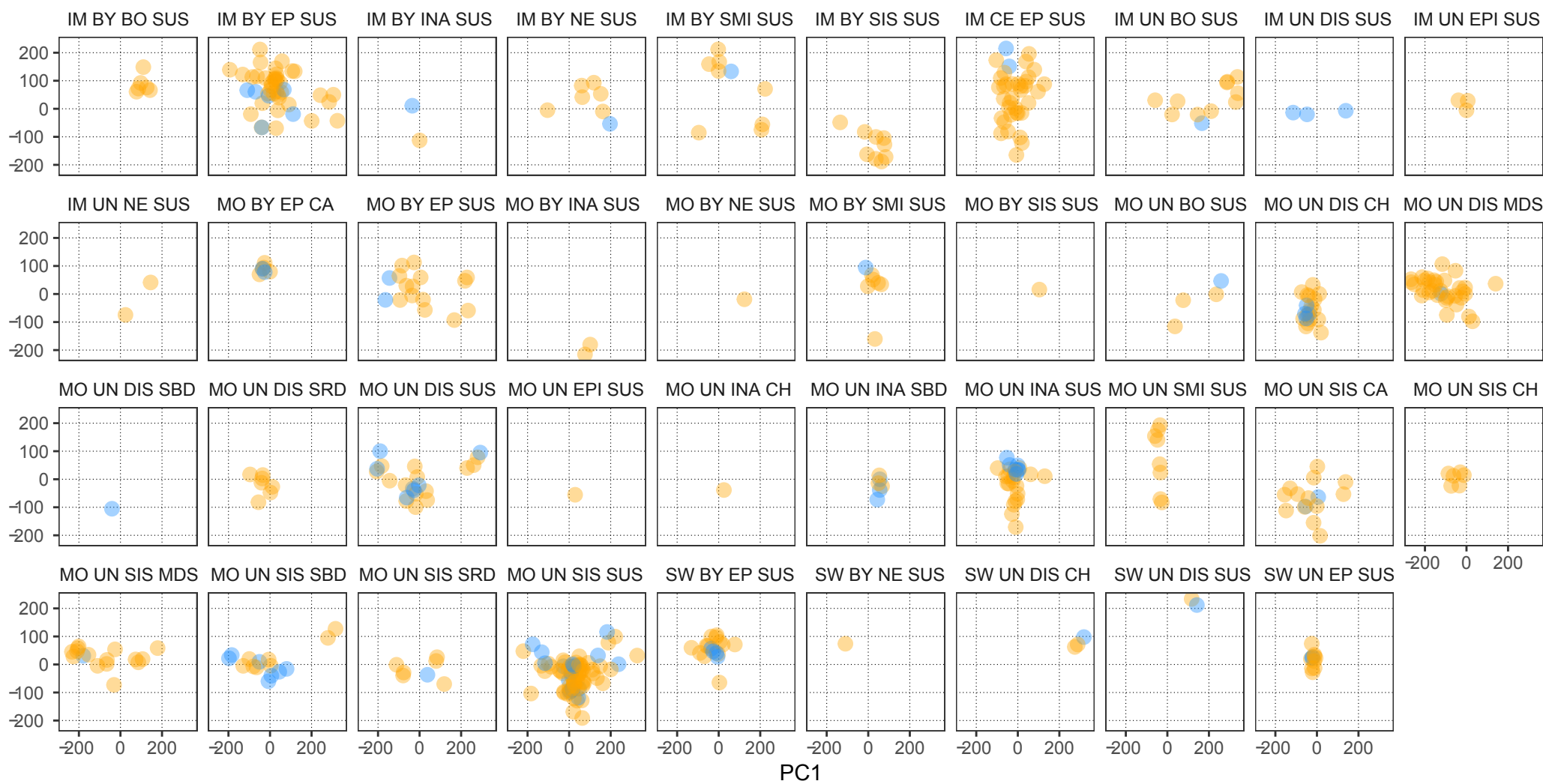

Supplement: S3 Fig — Multipanel figure displaying the same data as in Text Fig 3 but faceted by functional groups in order to clarify within-subgroup patterns. (PDF) [file pone.0221490.s005.pdf]

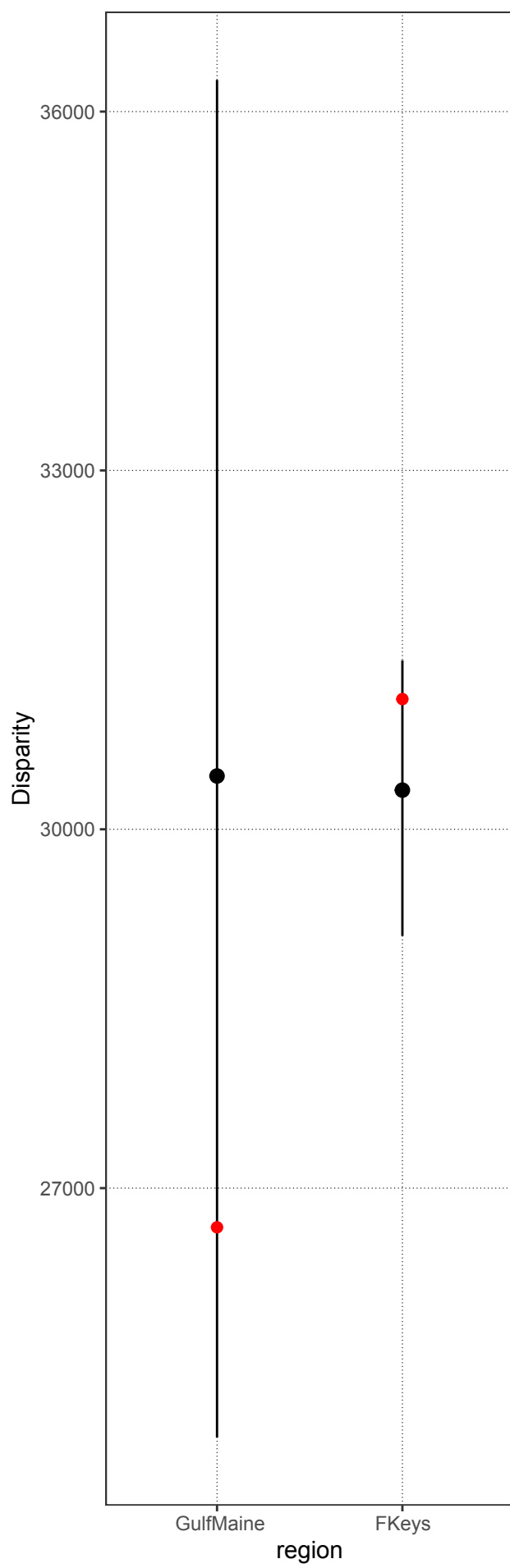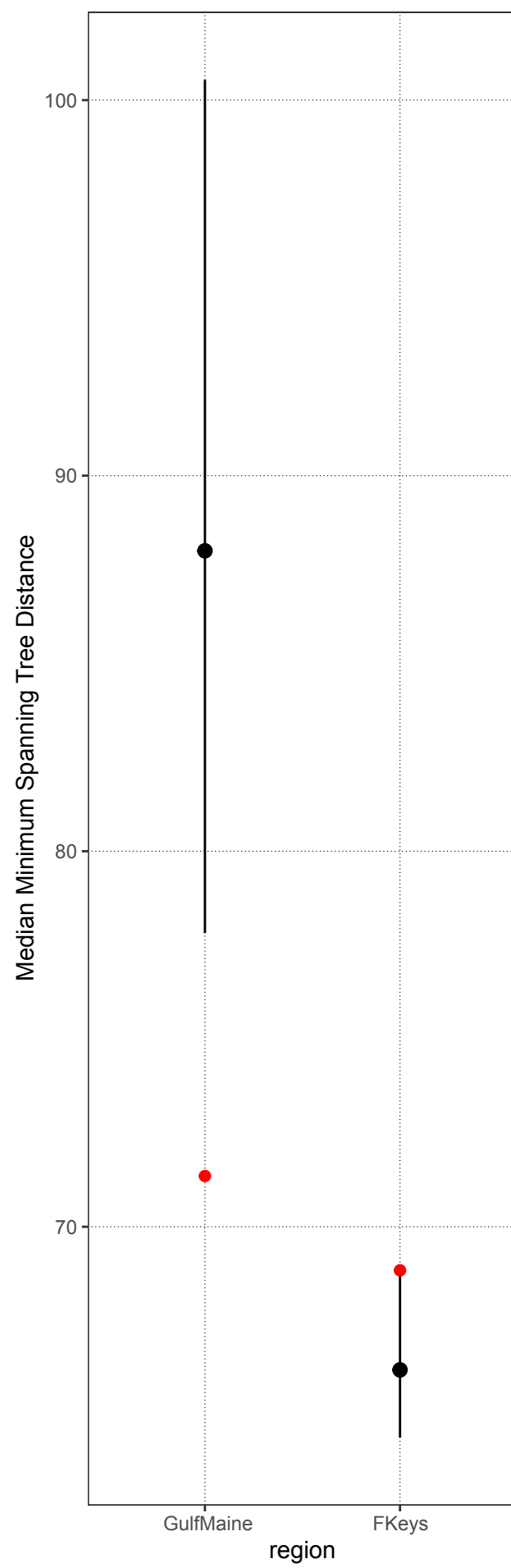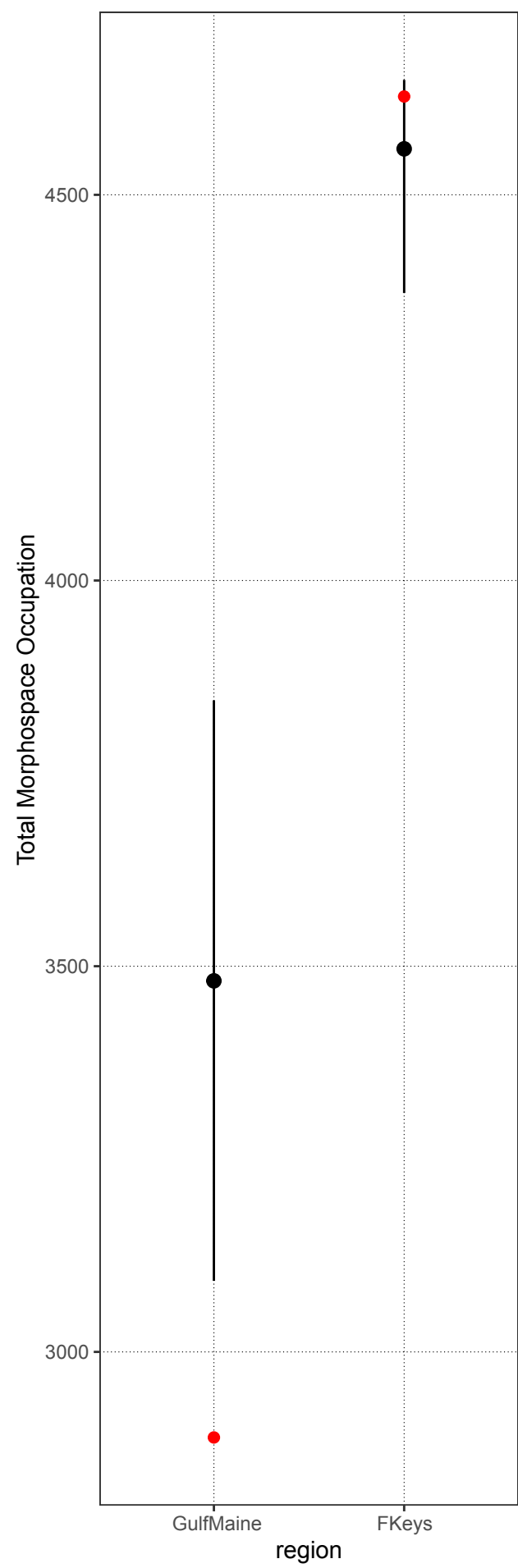

Supplement: S4 Fig — Faunal disparities, median minimum spanning tree distances, and total morphospace occupation for the whole faunas. Red points are observed values. Black points are the means and black lines are the 95% confidence intervals on the means of 1000 resampled sets. (PDF) [file pone.0221490.s006.pdf]

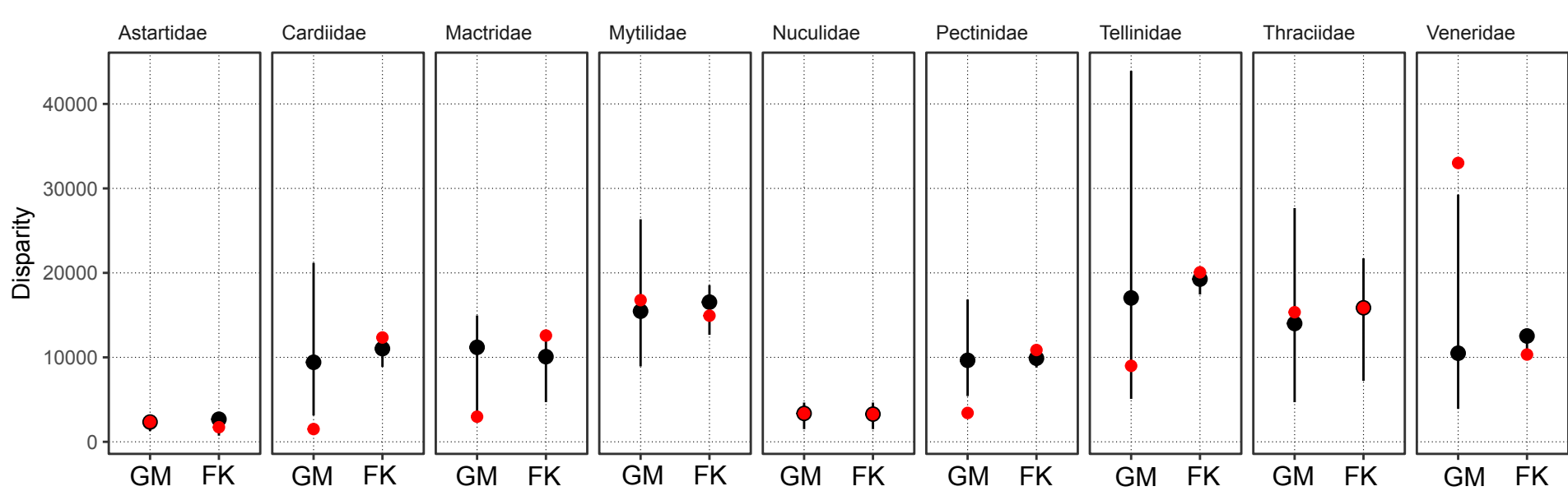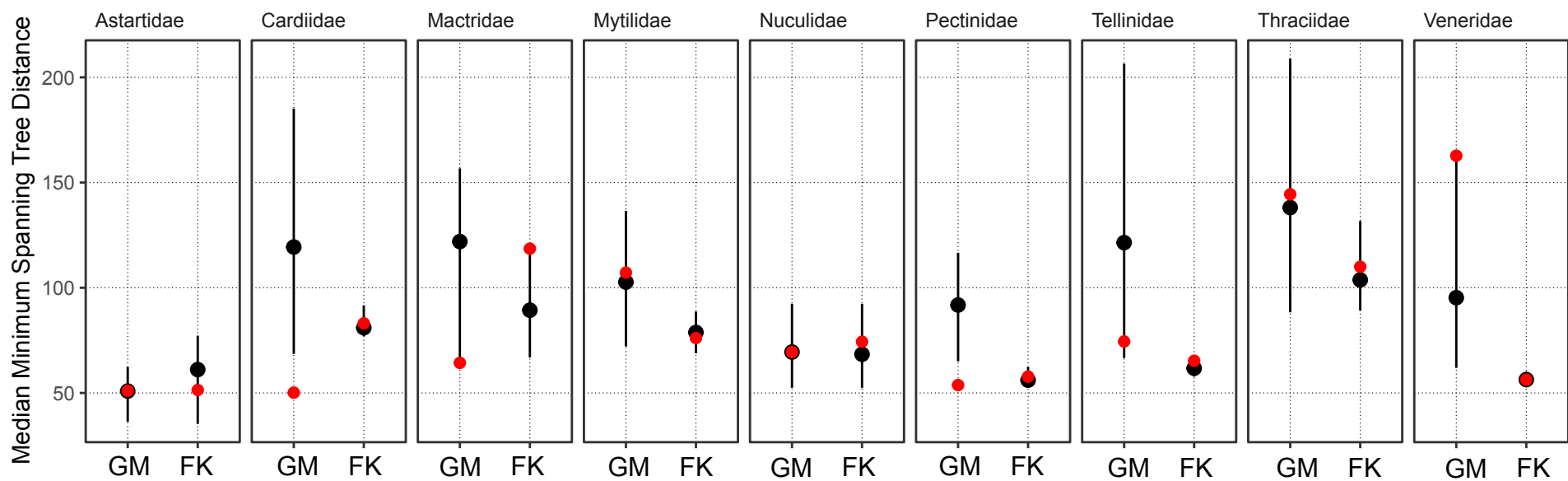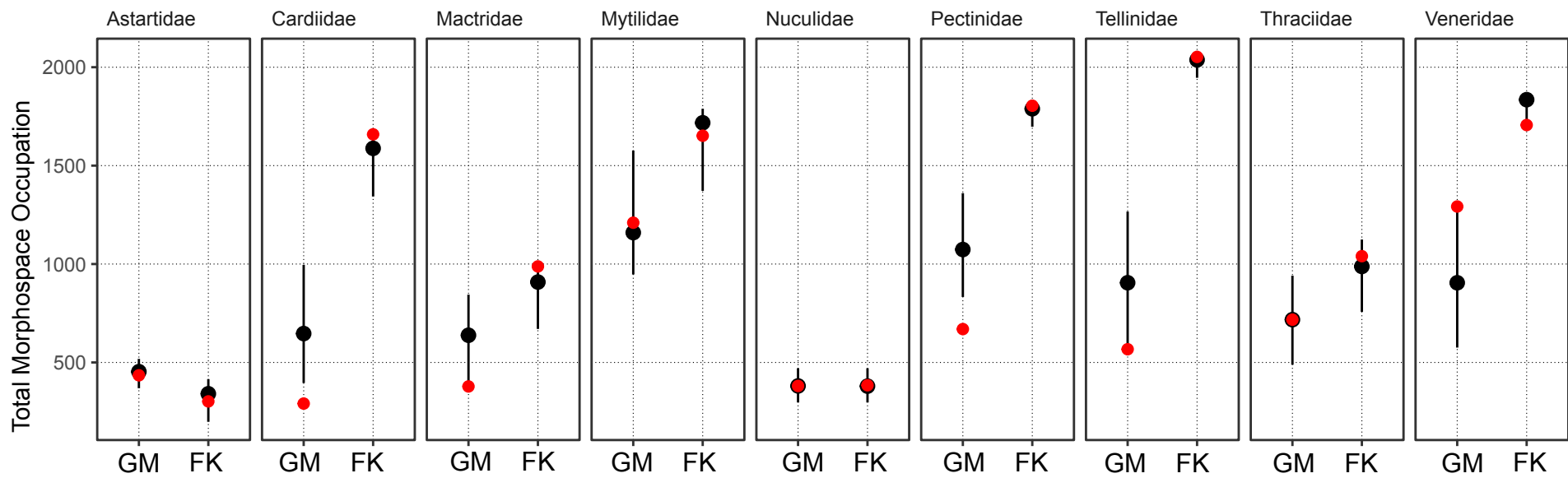

Supplement: S5 Fig — Faunal disparities, median minimum spanning tree distances, and total morphospace occupation for the families that are shared between regions. Red points are observed values. Black points are means and black lines are 95% confidence intervals on the means of 1000 resampled sets. (PDF) [file pone.0221490.s007.pdf]

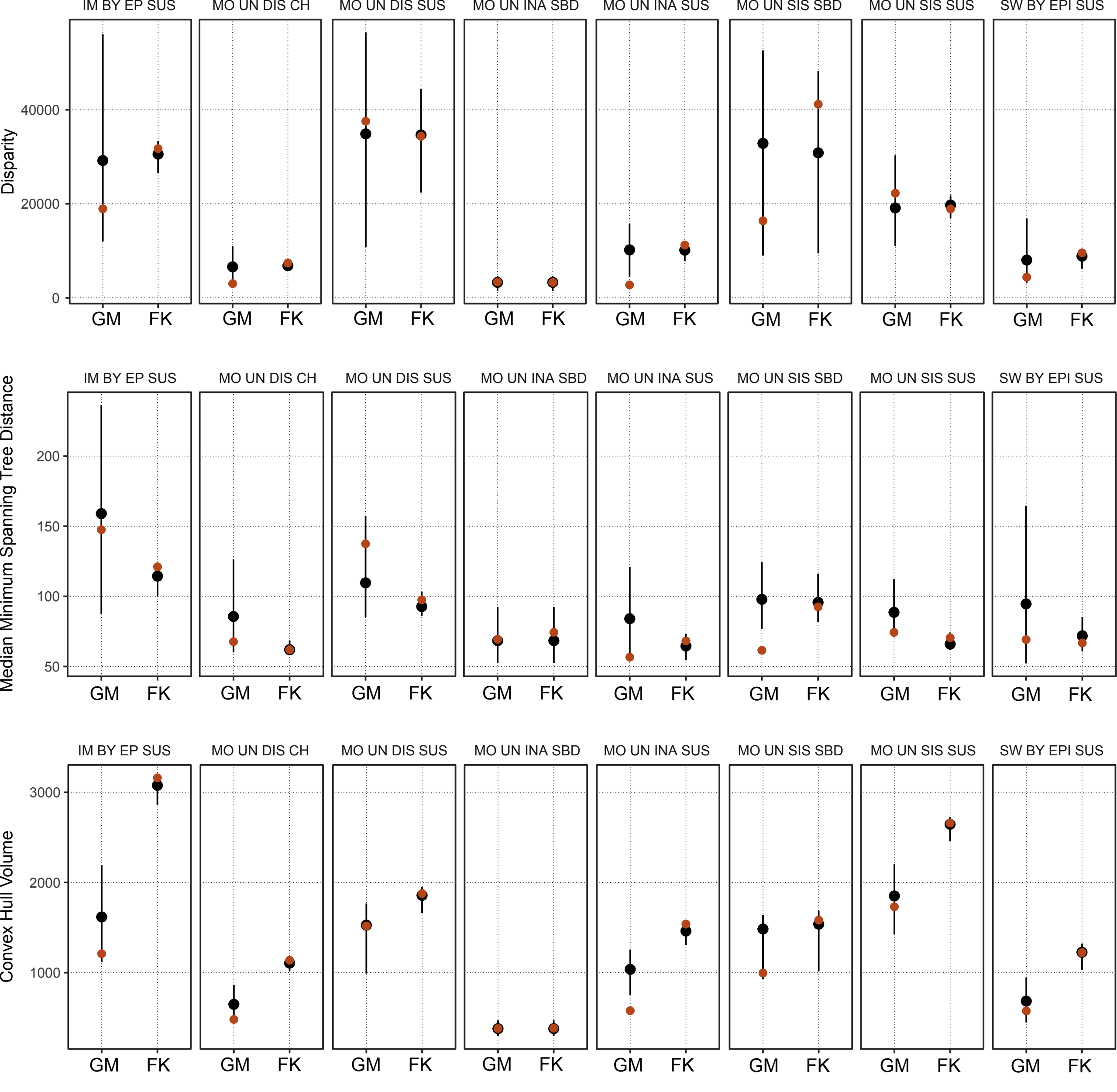

Supplement: S6 Fig — Faunal disparities, median minimum spanning tree distances, and total morphospace occupation for the functional groups that are shared between regions. Red points are observed values. Black points are means and black lines are 95% confidence intervals on the means of 1000 resampled sets. (PDF) [file pone.0221490.s008.pdf]

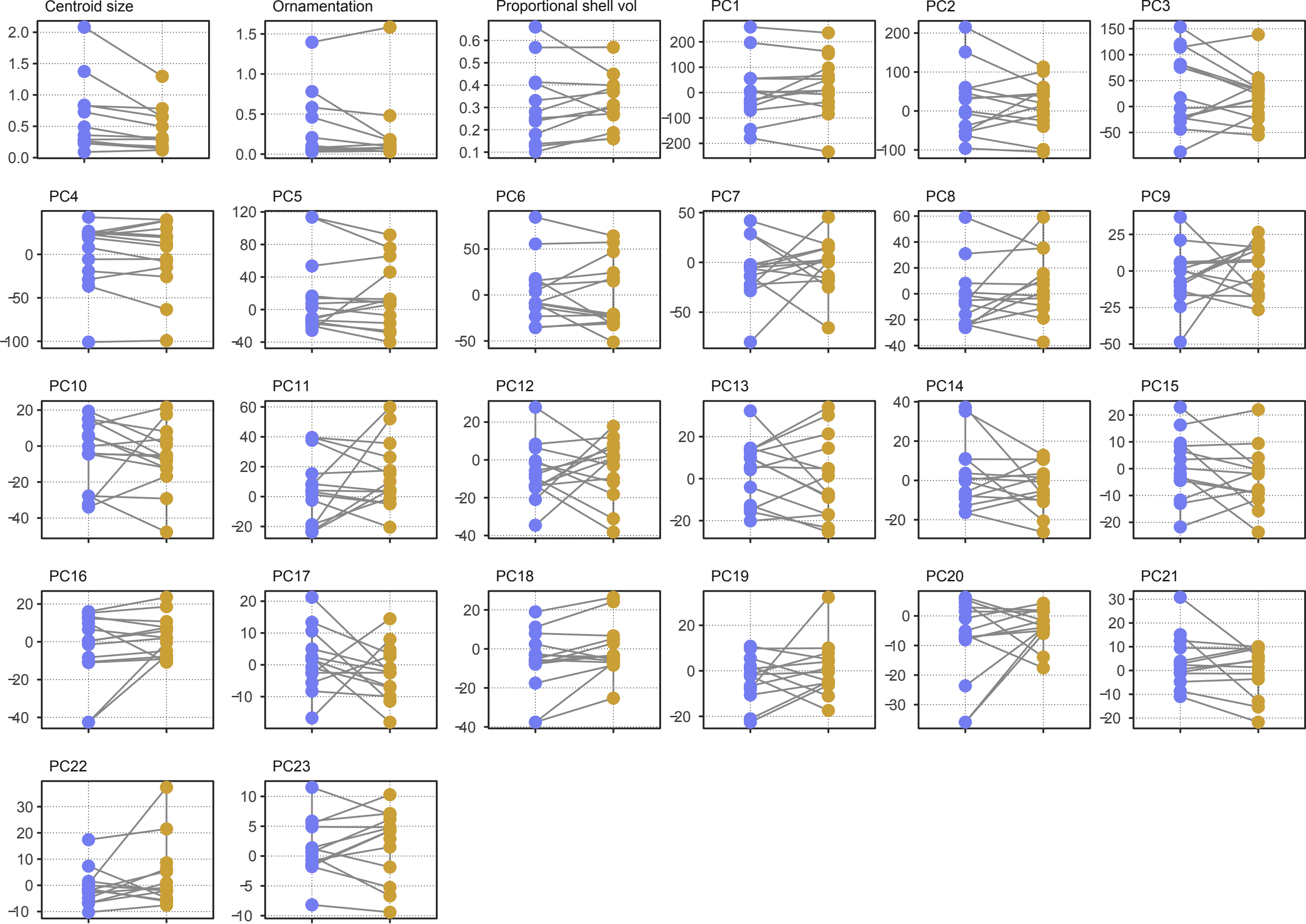

Supplement: S7 Fig — Blue points are GM specimens and orange points are FK specimens. Conspecific individuals are joined with grey lines (note that some specimens are represented by two valves in at least one region, hence grey lines occasionally join points within a region as well as between regions). Note that grey lines cross between regions, suggesting that there is no overarching latitudinal change within species. (PDF) [file pone.0221490.s009.pdf]

a Cumulative Sum

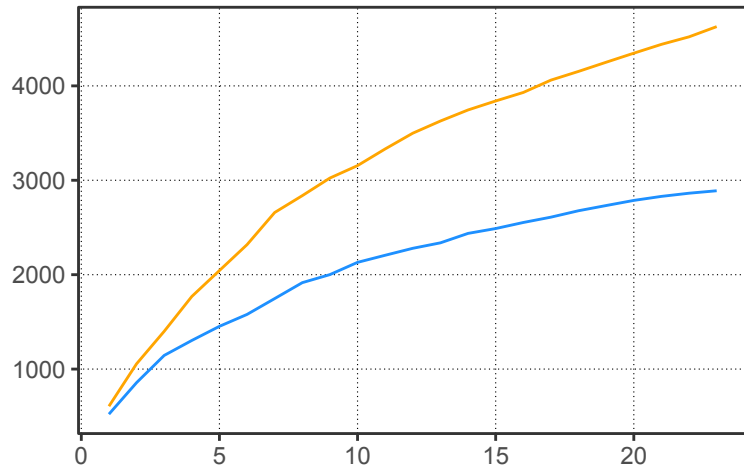

b Incremental Range

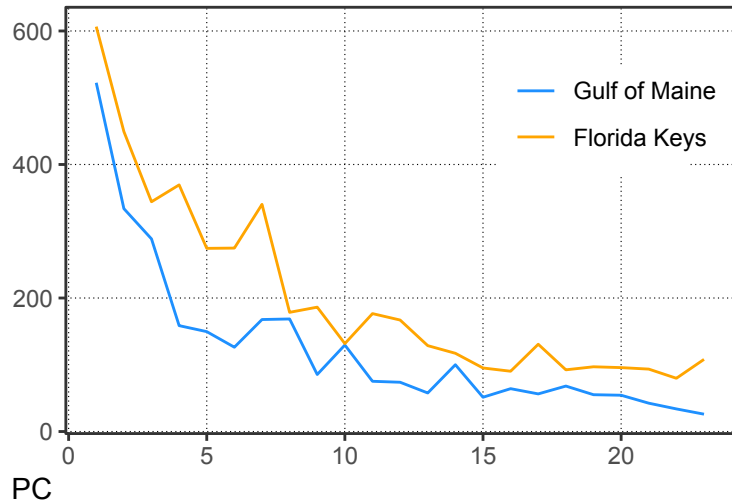

Supplement: S8 Fig — (a) The range of scores for each PC axis declines for both the Florida Keys and Gulf of Maine, but the Florida Keys consistently occupies greater range along each PC axis. (b) The cumulative sum of range values for each axis show that the Florida Keys occupies almost double the total morphospace of the Gulf of Maine across 23 PCs (FK = 4627, GM = 2889). (PDF) [file pone.0221490.s010.pdf]

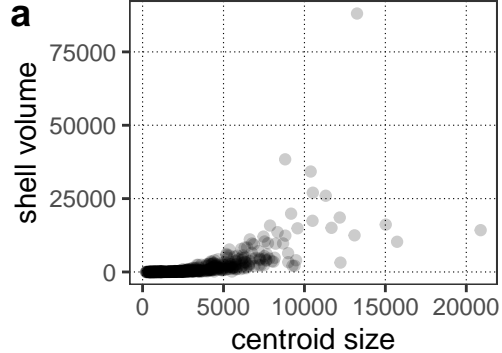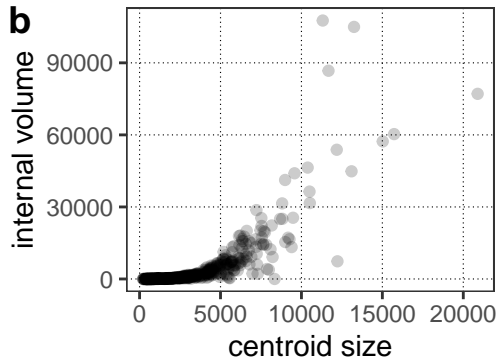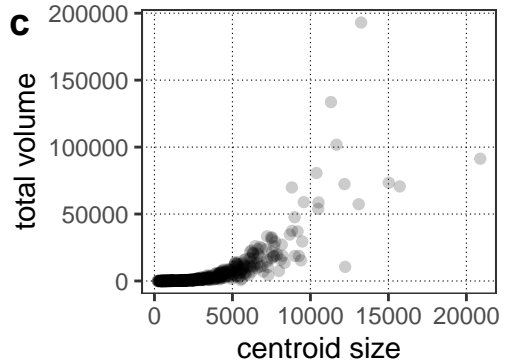

Supplement: S10 Fig — (a) shell volume (i.e. volume of body that is carbonate), (b) internal volume (i.e. soft-tissue+water volume), and (c) shell volume + internal volume. (PDF) [file pone.0221490.s012.pdf]
