# Supplementary material for: Spatial filters of function and phylogeny determine morphological disparity with latitude
Source: PLoS One. 2019 Aug 29;14(8):e0221490. doi: 10.1371/journal.pone.0221490 (PMC6715166; doi:10.1371/journal.pone.0221490)
Supplement: S9 Fig — (a) Root mean squared error between the true landmark configuration and a landmark configuration reconstructed using the specified subset PCs (i.e. those specified on the x-axis). Each black line represents one specimen. Box plots give the median, inner quartile, and inner 95% of values summarized across specimens at the specified PC. RMSE tends to stabilize by PC 20–25 (94–55% cumulative explained variance). (b) Location of all specimens along PCs 1–2, the specimens used to visualize the power analysis in panel c are highlighted in red. (c) Visualization of reconstructed shapes for specimens plotted in red in panel b. Species with greater shape complexities such as the posterior rostrum in Cardiomya striata require more PCs to faithfully reconstruct the general shell shape. Holes in the reconstructed meshes result from generating a mesh surface from reconstructed point-cloud and are not real features of the animal. (PDF) [file pone.0221490.s011.pdf]

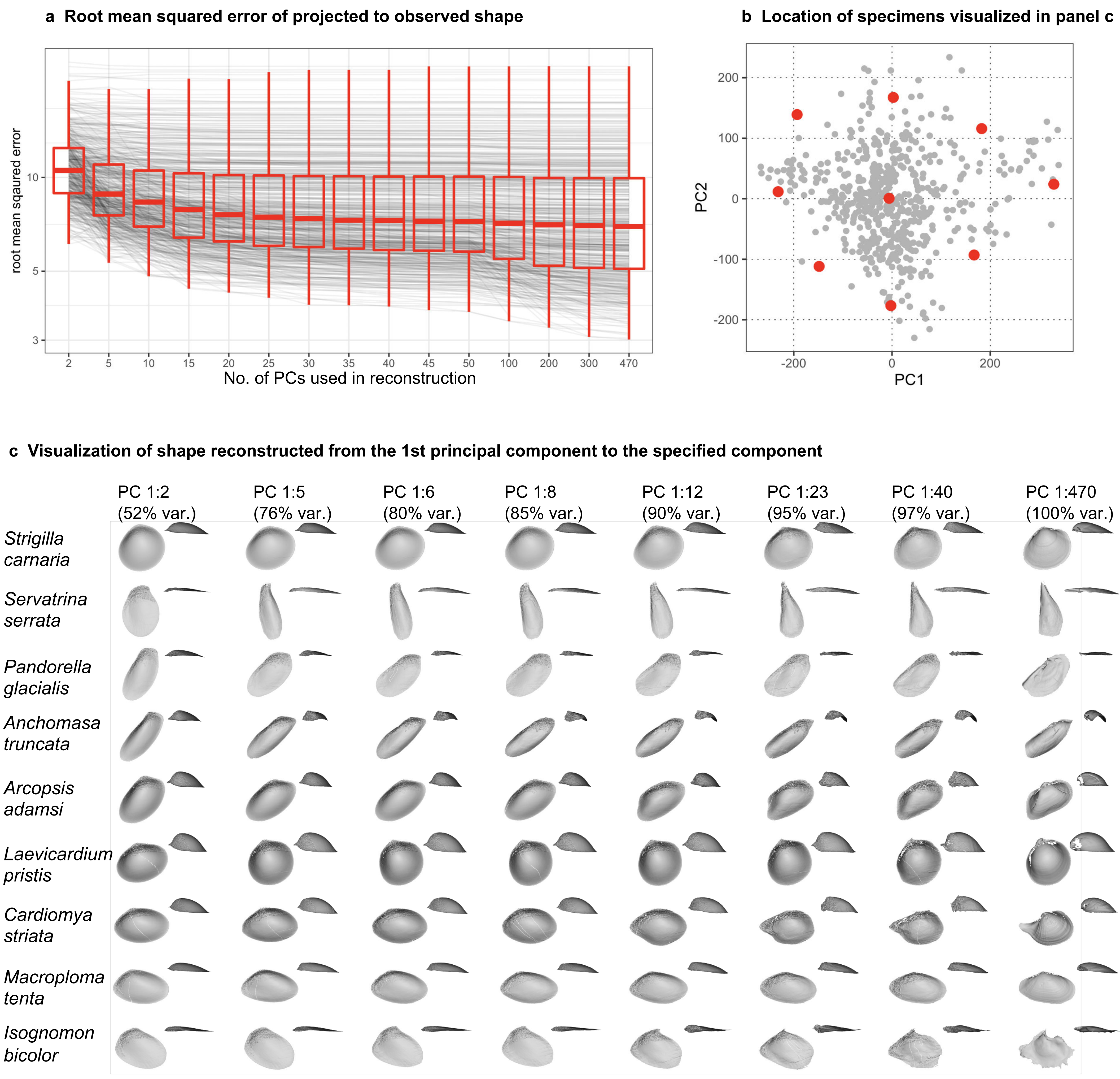
a Root mean squared error of projected to observed shape
root mean sqaured error
3
5
10
2
5
10
15
20
25
30
35
40
45
50
100
200
300
470
No. of PCs used in reconstruction
b Location of specimens visualized in panel c
PC2
200
100
0
-100
-200
-200
0
200
PC1
c Visualization of shape reconstructed from the 1st principal component to the specified component
PC 1:2 (52% var.)
PC 1:5 (76% var.)
PC 1:6 (80% var.)
PC 1:8 (85% var.)
PC 1:12 (90% var.)
PC 1:23 (95% var.)
PC 1:40 (97% var.)
PC 1:470 (100% var.)
Strigilla carnaria
Servatrina serrata
Pandorella glacialis
Anchomasa truncata
Arcopsis adamsi
Laevicardium pristis
Cardiomya striata
Macroploma tenta
Isognomon bicolor
